# Temporal transcriptomic analysis of the *Listeria monocytogenes *EGD-e σ^B ^regulon

**DOI:** 10.1186/1471-2180-8-20

**Published:** 2008-01-28

**Authors:** Torsten Hain, Hamid Hossain, Som S Chatterjee, Silke Machata, Ute Volk, Sandra Wagner, Benedikt Brors, Stefan Haas, Carsten T Kuenne, Andre Billion, Sonja Otten, Jan Pane-Farre, Susanne Engelmann, Trinad Chakraborty

**Affiliations:** 1 Institute for Medical Microbiology, Justus-Liebig-University, Frankfurter Strasse 107, D-35392 Giessen, Germany; 2 Div. Intell. Bioinformatics Systems, DKFZ, Im Neuenheimer Feld 280, D-69120 Heidelberg, Germany; 3 Department of Computational Molecular Biology, Max-Planck-Institute for Molecular Genetics, Ihnestrasse 73, D-14195 Berlin, Germany; 4 Ernst-Moritz-Arndt-University, Institute for Microbiology, Jahnstraße 15, D-17487 Greifswald, Germany

## Abstract

**Background:**

The opportunistic food-borne gram-positive pathogen *Listeria monocytogenes *can exist as a free-living microorganism in the environment and grow in the cytoplasm of vertebrate and invertebrate cells following infection. The general stress response, controlled by the alternative sigma factor, σ^B^, has an important role for bacterial survival both in the environment and during infection. We used quantitative real-time PCR analysis and immuno-blot analysis to examine σ^B ^expression during growth of *L. monocytogenes *EGD-e. Whole genome-based transcriptional profiling was used to identify σ^B^-dependent genes at different growth phases.

**Results:**

We detected 105 σ^B^-positively regulated genes and 111 genes which appeared to be under negative control of σ^B ^and validated 36 σ^B^-positively regulated genes *in vivo *using a reporter gene fusion system.

**Conclusion:**

Genes comprising the σ^B ^regulon encode solute transporters, novel cell-wall proteins, universal stress proteins, transcriptional regulators and include those involved in osmoregulation, carbon metabolism, ribosome- and envelope-function, as well as virulence and niche-specific survival genes such as those involved in bile resistance and exclusion. Ten of the σ^B^-positively regulated genes of *L. monocytogenes *are absent in *L. innocua*. A total of 75 σ^B^-positively regulated listerial genes had homologs in *B. subtilis*, but only 33 have been previously described as being σ^B^-regulated in *B. subtilis *even though both species share a highly conserved σ^B^-dependent consensus sequence. A low overlap of genes may reflects adaptation of these bacteria to their respective environmental conditions.

## Background

Stress responses in bacteria are mediated by alternative sigma factors and other regulatory proteins. In the gram-negative bacterium *E. coli*, extensive studies have identified the *rpoS *gene as the factor orchestrating cellular responses to stressful conditions [1,2]. In the low G+C content, gram-positive genera like *Bacillus*, *Listeria *and *Staphylococcus *the alternative sigma factor, σ^B^, is involved in coordinating cellular stress responses [3-8]. In *B. subtilis*, members of the σ^B ^regulon contribute to the microorganism's response to environmental stress conditions such as starvation in stationary growth phase, heat, osmotic stress, repeated freezing and thawing or acid and alkaline shock [9-11]. More than 100 stress-responsive genes have been assigned to be under the control of the σ^B ^transcription factor [12,13].

The activity of σ^B ^in *B. subtilis *is known to be post-translationally regulated by a complex network composed of two sequentially linked partner-switching modules [14]. The *sigB *operon consists of *sigB *itself and seven regulators of *sigB *(*rsb*) genes. These regulatory genes can either negatively (e.g. *rsbW*, *rsbX*) or positively control σ^B ^activity (e.g. *rsbV*). Under normal conditions σ^B ^is inactivated by direct binding by the anti-σ^B ^protein RsbW. Induction of the σ^B ^regulon is effected by sequestration of the RsbW protein (anti-σ^B^) by RsbV (anti-anti-σ^B^) thus releasing of the previously complexed σ^B ^protein, which then forms the holoenzyme with the core RNA polymerase to transcribe genes with σ^B^-dependent specificities [7,14,15].

Comparative analysis reveals that there is considerable variation in the genome organisation of genes comprising *sigB *operons in *B. subtilis *and other gram-positive bacteria [16,17]. The overall organization of genes is best conserved between *L. monocytogenes*, *L. innocua *and *B. subtilis *but truncated versions of the *sigB *operon lacking the *rsbR*, *rsbS*, and *rsbT *are found in *B. anthracis*, *S. aureus *and *S. epidermidis*. Given that these bacteria occupy different ecological niches it would be interesting to examine the extent of conservation of the σ^B ^regulons amongst these bacteria.

*L. monocytogenes *is a ubiquitous gram-positive, facultative intracellular bacterium causing severe disease in both animals and humans. *L. monocytogenes *is well-known for its robust physiology. It is capable of growth at refrigeration temperature, low pH and at high osmolarity. However, relatively little is known about the role of adaptive physiological response in promoting survival and growth of the bacterium in hostile environments. Several studies demonstrated that a *L. monocytogenes sigB *deletion mutant was more susceptible to environmental stresses such as acid stress [18], osmotic stress [3,19] and carbon starvation [8,19]. A strong σ^B^-dependence has been shown for the osmotic induction of both *opuC *(encoding an ABC carnitine transporter) and *lmo1421 *(encoding a putative transporter) [20], and for one of the two *prfA *promoters upstream of *prfA *[21]. Furthermore, a deletion in the *sigB *gene has been reported to affect the virulence phenotype of *L. monocytogenes *in murine and guinea pig models of infection [21,22]. Recently, 55 σ^B^-regulated genes have been identified using a customized 208-gene microarray comprising of genes predicted to harbor σ^B^-dependent promoters [23].

Even though entry into stationary phase in BHI medium is known to induce σ^B ^activity, the exact nature of the inducing signal remains unknown. In this study we monitor σ^B ^expression at different stages of growth using quantitative real-time PCR analysis (qRT-PCR) and immuno-blot analysis at different stages of growth. We examined for genes of the *sigB *regulon expressed at different bacterial growth phases using whole-genome based transcriptome profiling. A total of 216 σ^B^-dependent genes of which 105 were positively and 111 genes negatively regulated were detected in the presence of the alternative sigma factor σ^B^.

## Results and Discussion

Using temporal transcriptome profiling, we identified 216 genes in *L. monocytogenes *EGD-e as being differentially regulated by the alternative sigma factor, σ^B^, at four different time points of bacterial growth (3 h, 4 h, 8 h and 16 h) in brain heart infusion (BHI) broth. These correspond to middle and late exponential phases (3 h and 4 h) as well as early and late time points of stationary phases (8 h and 16 h) of growth, respectively (see Additional file 1, Fig. 4S).

### Analysis of the σ^B^-expression during bacterial growth

**Figure 1:**
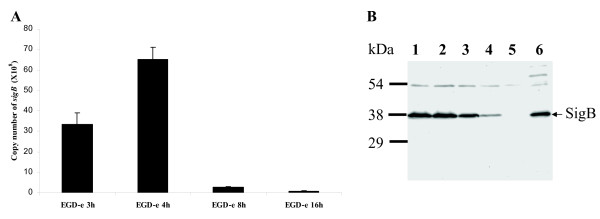
(A) Copy number of the *sigB *gene in *L. monocytogenes *EGD-e grown in BHI medium for 3 h, 4 h, 8 h and 16 h. Data shown here is representative of three independent biological replicates. (B) Immuno-blot analysis quantifying σ^B ^from *L. monocytogenes *EGD-e at different growth phases. Proteins were isolated from cultures of *L. monocytogenes *EGD-e grown for 3 h (lane 1), 4 h (lane 2), 8 h (lane 3) and 16 h (lane 4) in BHI at 37°C and σ^B ^was detected using rabbit polyclonal anti-serum produced against *S. aureus *COL σ^B^. The *L. monocytogenes *Δ*sigB *deletion mutant (lane 5) was used as negative control and *S. aureus *COL (lane 6) was used as positive control for specific binding of the antibody. Molecular masses (in kilodaltons) of prestained SDS-PAGE standard marker (Bio-Rad) are indicated on the left and σ^B ^is marked on the right.

We assayed σ^B ^expression at the various bacterial growth phases and found that its expression is highly induced at 3 h and 4 h both by qRT-PCR (Fig. 1A) and by monitoring protein expression levels using a σ^B^-specific polyclonal antibody (Pane-Farre *et al*., manuscript in preparation) in immuno-blot analysis (Fig. 1B). Expression of the σ^B ^regulatory protein decreases at the 8 h time point and is lowest at 16 h, the last time point examined in this study (Fig. 1). This expression pattern suggests that in *L. monocytogenes*, σ^B ^probably reflects a requirement in regulating responses to environmental changes during rapid growth.

### Monitoring σ^B^-regulated genes using a reporter gene fusion assay

To demonstrate that σ^B ^acts as a transcriptional activator we examined the σ^B^-dependence of genes seen in the microarray studies by cloning upstream regions from 41 σ^B^-up regulated genes and fusing them to a promoterless β-galactosidase gene (*bgaB*) derived from *B. stearothermophilus *on plasmid pSOG30222 (see Additional file 1, Fig. 2S). Of these 41 genes, 33 promoters harbored canonical σ^B^-binding sequences while 8 had no putative σ^B^-binding sequences but were nevertheless considered to be σ^B^-regulated on the basis of transcriptome analysis. We verified the dependence of the promoter regions on σ^B ^by conducting gene fusion assays. Under the growth conditions described in Material and Methods, we found between 2- and 230-fold more activity in the wild type as compared to the Δ*sigB *mutant strain.

Five promoters with putative σ^B^-binding sequences were slightly activated by σ^B^, but did not fulfill the criteria for being σ^B^-dependent (*lmo0043*, *lmo1433*, *lmo2067*, *lmo2231 *and *lmo2485*). In summary, out of 41 promoters tested, we confirmed 36 promoters (~88%) for σ^B^-dependency (see Additional file 1, Table 1S).

### Reevaluation of the consensus binding site for σ^B^

Based on the results presented here we infer that the σ^B ^regulon comprises 216 transcription units. Using the pattern GTTT-N_12–17_-GGGWAW for σ^B^-binding motif search, we investigated the upstream sequences of the putative 216 σ^B^-dependent genes and were able to detect σ^B^-binding motifs in 68 of the 216 upstream sequences inspected (see Additional file 1, Table 1S and Table 2S).

The analysis successfully identified 35 of 54 previously known σ^B^-binding sites [23]. A high proportion of predicted σ^B^-binding sites were located upstream of the gene, but in five cases predicted sites were located within the coding sequence (*lmo0911*, *lmo0994*, *lmo2463*, *lmo2485 *and *lmo2724*).

105 genes were positively controlled by σ^B ^and correspond to 19 operons, encoding 48 genes as well as 57 single-gene transcription units (see Additional file 1, Table 1S). Of these 76 transcription units, 51 harbor a single consensus σ^B^-binding sequence, 2 transcriptional units have two σ^B^-binding sequences each and the remaining 23 do not contain consensus σ^B^-binding sequences. These are likely to encode gene products whose expressions are probably either controlled by σ^B^-dependent regulatory genes or whose RNA is stabilized by RNA binding proteins whose activities are σ^B^-regulated such as Hfq.

Of the 111 genes down regulated during σ^B ^expression, 28 genes were organized in 11 operons and 83 were single genes. We identified only 13 consensus σ^B^-binding sequences among the 111 σ^B^-down regulated genes (see Additional file 1, Table 2S). σ^B^-dependent down regulated genes were solely found in both the stationary phase growth conditions tested, but were not detected in bacteria at the exponential growth phase. It is likely that σ^B ^regulates expression of genes that negatively affect (regulate) the central metabolism of the cells, as the cells move away from active growth.

### Chromosomal distribution of the σ^B^-dependent genes

**Figure 2:**
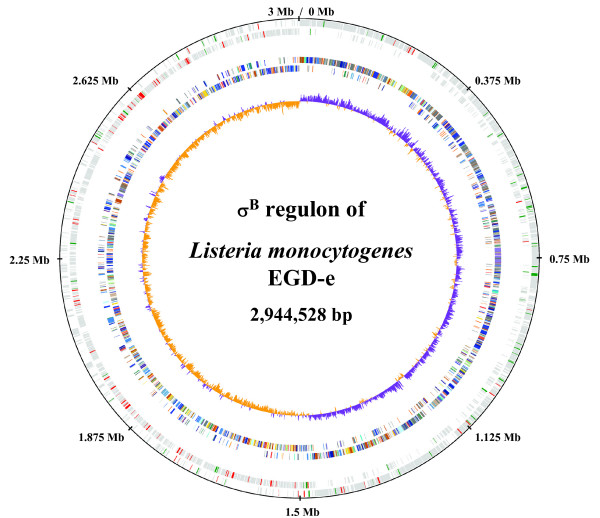
Chromosomal distribution and orientation of σ^B^-differential regulated genes. The first circle represents the scale in kb starting with the origin of replication at position 0. The second circle shows the distribution of coding sequences (CDS) of the leading and lagging strand in gray including σ^B^-dependent up regulated genes in green and σ^B^-dependent down regulated genes in red. The third circle indicates the COG classification for each gene and distribution of COG classes within the genome. Colour scheme and categories were chosen to the convention of the COG database. The innermost circle displays the GC skew ([G+C]/[G-C]), with values greater than zero in purple and less than zero in orange. For GC skew analysis we applied a sliding window of 1000 bp with an overlap of 500 bp. The figure was drawn using the GenomeViz software [70].

Our studies suggest that genes comprising about 7.6% of the genome of *L. monocytogenes *are regulated directly or indirectly by σ^B^. Chromosomal distribution of the σ^B^-dependent genes revealed an uneven distribution over the genome of *L. monocytogenes *(Fig. 2). 72% of the σ^B^-positively regulated genes (76 of 105 genes) were located on the first third of each replicore. It has been suggested that expression of genes located near the *oriC *is higher in growing cells compared to genes located around the terminus [24]. Interestingly, the majority of σ^B^-negatively controlled genes (82%) were concentrated on the left replicore.

### Functional classification of the σ^B^-regulon

**Figure 3:**
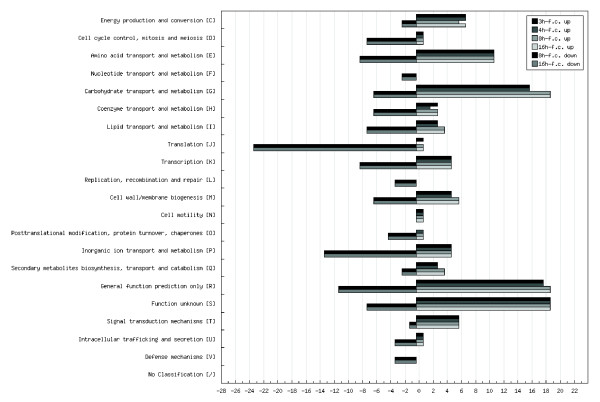
Functional COG categories of σ^B^-differential regulated genes of *L. monocytogenes *EGD-e obtained from temporal transcriptome profiling experiments. Functional categories were determined using COG for *L. monocytogenes *provided by NCBI [62]. The figure was drawn using the Augur software [71].

Considering the candidate genes identified in our study, the σ^B ^regulon in *L. monocytogenes *comprises genes involved in metabolic functions including glucose and amino acid metabolism, solute transporters, permeases, ABC transport systems, general stress proteins, cell wall proteins, bile resistance and exclusion and transcription factors (Fig. 3). The total number of σ^B^-regulated genes might be underestimated since the transcriptional activity of the strain was monitored only under growth conditions in complex BHI media during the middle and late exponential and the early and late stationary phase. In a similar study that examined the size of the regulon controlled by the master regulator of virulence PrfA in strain EGD-e, Milohanic and colleagues [25] reported a subcategory of 53 genes harboring σ^B^-like binding sequences in their respective promoter regions. Here, we confirm that 45 of those 53 genes (85%) are indeed σ^B^-regulated genes. An earlier study reported 55 σ^B^-regulated genes identified during growth in the stationary phase (using BHI) and under salt stress [23]. Only 35 of the 53 (64%) genes of that study were detected here. However, that study employed a different strain 10403S and used sequences derived from the genome sequence of strain EGD-e as the basis for the design of the array.

In ongoing studies, we examined the global transcriptional response after different shock conditions for *L. monocytogenes *[26] and found 92 heat stress induced genes all of which are present among the 105 σ^B^-positively regulated genes found in this study. This suggests the potent role of σ^B ^in growth under hostile environmental conditions.

### Osmolarity, bile and acid response

Several genes, including the OpuC, ABC-like transporter (*lmo1425-1428*) that are linked to choline, glycine-betaine and carnitine uptake, have been shown to contribute to protection against osmotic stress in *L. monocytogenes *[27]. In agreement with these results, we confirmed the presence of a σ^B^-binding sequence and strong σ^B^-dependent activity in the promoter region preceding the *opuCA *(*lmo1428*) gene [20,27]. We also found that genes coding for a transport system for di- and tripeptides, DtpT (*lmo0555*) [28] and a peptidase T (*lmo1375*), were positively regulated by σ^B^. Degradation of peptides to their constituent amino acids can serve either as osmoprotectants (glycine, proline), or substrates for other amino acids or even as a source of energy.

A member of ornithine cycle, a putative arginine deiminase (*lmo0043*) based on sequence homology, known to catalyse the conversion of L-arginine to L-citrulline and ammonia was observed to be under σ^B ^control. The ammonia metabolized probably serves as a protectant in other bacteria against acid damage [29], and the citrulline produced might be used to generate ornithine.

Further a operon comprising the genes for *lmo1421 *and *lmo1422 *encoding the bile exclusion system BilE was confirmed to be σ^B^-regulated [30]. Together with the bile salt hydrolase *bsh *(*lmo2067*) [31], we identified three genes that contribute to neutralization of bile in the gastrointestinal tract.

In this study, we confirmed the extreme sensitivity of the Δ*sigB *mutant to low acid conditions [18] and bile [32] (see Additional file 1, Fig. 1S and Fig. 3S). The presence of specific genes for mediating bile- and acid-resistance in *L. monocytogenes *probably reflects niche-specific adaptation of this species to colonize the gastrointestinal tract of mammalian hosts.

### Carbohydrate metabolism

The largest set of hitherto undescribed σ^B^-dependent genes is linked to the biosynthesis of carbohydrates. Thus σ^B^-dependent activation of genes encode putative glucose, mannose- and fructose specific uptake systems (*lmo0169*, *lmo0781-784*) are coupled to the up regulation of several enzymes involved in glycolysis including two dihydroxykinases and a dihydroxyacetone kinase phosphotransfer protein (*lmo2695-2697*) as well as phosphoglycerate mutase (*lmo2205*).

Furthermore, genes coding for NagA (*lmo0956*) and NagB (*lmo0957*) are involved in the cell wall metabolism leading to the generation of glucosamine-6-phosphate via acetylglucosamine-6-P-deacetylase (NagA) and further on to fructose-6-phosphate using NagB, a glucosamine-6-phosphate isomerase [33]. Fructose-6-phosphate can act as initial precursor for the glycolysis pathway. It can be suggested that use of cell wall components such as aminosugar supports metabolism during stress situations, when other energy rich compounds are not accessible.

Genes encode enzymes of the pentose phosphate pathway including glucose-1-dehydrogenase (*lmo0669*) as well as the ribose-5-phosphate isomerases (*lmo2674 *and *lmo0736*) and a ribulose-phosphate-3-epimerase (*lmo0735*) show σ^B^-dependent regulation. The ORF *lmo0736 *belongs to the group of genes without a homolog in *L. innocua*. Comparative genome analysis including the recently published *L. monocytogenes *strains [34,35] showed that both *lmo0735 *and *lmo0736 *lie within a region that is specific to *L. monocytogenes*.

In addition, we found components of pyruvate metabolism pathway to be induced, as seen by the activation of a pyruvate oxidase (*lmo0722*) and an acetate kinase (*lmo1168*) that can generate acetyl-CoA to enter into the tricarboxylic acid (TCA) cycle. Finally, we identified two putative NAD-dependent dehydrogenases (*lmo0794 *and *lmo2391*) and a NADPH-dependent quinone reductase (*lmo2573*), both of which were highly up regulated and are probably involved in electron transport and generation of cellular energy for the bacterial cell.

A functional overview of σ^B^-up regulated genes is given in Fig. 3 indicating a strong preference of categories for carbohydrate/amino acid transport and metabolism as well as for cell wall/membrane biogenesis.

### Transport of inorganic ions

Several transport processes for a range of inorganic ions in *L. monocytogenes *are under σ^B ^control. The gene encoding *lmo0405 *resembles low affinity phosphate transporter *pitA *of *E. coli *which is involved in Zn^2+ ^efflux when the ion reaches highly toxic external concentrations [36]. In addition to phosphate uptake, the *phoU *gene (*lmo2494*) encoding a negative regulator of the phosphate regulon was induced by σ^B^.

To regulate the concentration of heavy metal cations such as cadmium, zinc or cobalt, the bacterium might uses another putative transporter *lmo2231 *that has similarities to CzcD, a proton-driven antiporter system for these divalent cations [37]. The ORF encoded by *lmo2602 *is similar to MgtC of *M. tuberculosis *and *S. typhimurium*. MgtC is reported to support bacterial intracellular survival within the macrophages and increase the ability to grow in low Mg^2+ ^environments [38].

### Universal and general stress related genes

All three members of the universal stress proteins (Usp) of *L. monocytogenes*, namely *lmo0515*, *lmo1580 *and *lmo2673 *are up regulated by σ^B^. Using two dimensional gel electrophoresis, Lmo1580 was recently identified, a protein which is induced during acid adaptation and acid stress in *L. monocytogenes *EGD-e [39]. In *E. coli*, Usps are proteins that accumulate in cells during stationary phase and during a variety of stress conditions (heat shock, ultraviolet light, ethanol stress etc.) causing growth arrest in cells. Furthermore, Usp proteins are required for the management of DNA damage and are induced by mutations in the FtsK protein, a cell division protein located at the cell septa and which coordinates chromosomal dimer segregation during cell division resulting in the separation of the daughter cells. Here we demonstrate that in *L. monocytogenes*, FtsK (*lmo1606*) expression is also σ^B^-dependent, suggesting coordinated expression with Usps to prevent chromosomal damage during cell separation.

Genes regulated by the σ^B ^regulon mediating resistance to oxidative stress include a glutathione reductase encoded by *lmo1433*. For *L. lactis *it was shown that accumulation of intracellular glutathione conferred resistance to H_2_O_2 _[40]. We also observed and confirmed that the expression of Hfq (*lmo1295*) to be dependent on σ^B ^in *L. monocytogenes *[41]. The homolog protein in *E. coli *interacts with many regulatory sRNAs increasing e.g. the stability of the sRNA itself.

Recently, it was demonstrated that a Δ*hfq *mutant in *L. monocytogenes *EGD-e is sensitive to osmotic and ethanol stress, and is required for the expression of full virulence in the mouse model of infection [41]. Furthermore, co-immunoprecipitation using Hfq-specific antiserum followed by enzymatic RNA sequencing revealed that Hfq is able to bind different sRNA molecules [42].

### Virulence

Previously, Nadon and colleagues [21] provided evidence for σ^B^-dependent transcription of the listerial *prfA *gene (positive regulatory factor A) that controls the expression of all known virulence genes. Considerable similarity was found between the second promoter sequence of *prfA *(*prfAp*_2_) 5'-TTGTTACT-N_14_-GGGTAT-3' and the consensus σ^B^-binding sequence. To support their hypothesis, Nadon and coworkers [21] compared the phenotypes of a Δ*prfAp*_1_Δ*prfAp*_2 _and a Δ*sigB*Δ*prfAp*_1 _double mutant and showed that the loss of σ^B ^activity had the same effects as deletion of the *prfAp*_2 _promoter. As a result, expression of well-characterized PrfA-dependent virulence genes such as listeriolysin is induced under stress conditions[21]. Furthermore, *prfAp*_2 _was recently confirmed to be under direct control of σ^B ^in a growth phase dependent manner [43].

In this study, we were unable to demonstrate significant expression of *inlAB *genes, whose expression is PrfA-dependent. It has been previously reported that the Δ*sigB *mutant [23] is affected in epithelial cell invasion [22,44,45] as well as in gastrointestinal infection of guinea pigs [22]. Here we detected a number of genes predicted to encode cell wall-associated proteins that may adhere to collagen (*lmo0880 *and *lmo2085*), two other LRR-containing internalin proteins (*lmo0263 *and *lmo0610*) and predicted cell membrane proteins (*lmo0591*, *lmo1261*, *lmo2484 *and *lmo2602*) all of which are potential candidates for epithelial cell invasion. We also noted that the *bsh *and *bilEAB *genes whose transcription was previously shown to be PrfA-dependent [30,46], were significantly regulated (see Additional file 1, Table 1S) by σ^B ^as previously reported [31,47]. The data suggested strain-dependent differences in responses to various stress stimuli in the various *L. monocytogenes *strains, an observation that has previously been documented [48].

### Putative σ^B^-down regulated genes in stationary phase growth

Analysis of the expression profiles deriving from the early and late stationary growth phase revealed 111 genes down regulated by σ^B^. COG analysis of σ^B^-repressed genes (Fig. 3) included different categories which are e.g. involved in translation (initiation factor *infC*, elongation factor *tsf*, ribosome recycling factor *frr*, several ribosomal genes: *rpsT*, *rpmA*, *rplU*, *rpsD*, *rpsI*, *rplM*, *rpsH*, *rplN*, *rpsC*, *rplV*, *rpsS*, *rplB *and *rplD *and t-RNA synthetases: *lysS*, *alaS *and *hisS*). Other categories of repressed genes were involved in cell cycle control (*smc*), cell division (*ftsH*, *ftsW ftsZ*, *ftsQ*, *ftsI*, *ftsL*, *ftsX*, *ftsE *and cell division inhibitor *minD*), cell wall biogenesis (*mreD*, *iap *and *spl*) and inorganic ion transport for manganese, copper and cobalt (*mntH*, *mntBCA*, *lmo2062 *and *cbiQ*) (Fig. 3 and see Additional file 1, Table 2S). σ^B ^repression of ribosomal genes *rlpD*, *rpsC*, *rplW *and *rpsJ *and genes encoding cell wall lytic enzymes as well as ion binding proteins was previously reported for *B. subtilis *[13].

Here we report that σ^B ^regulates many aspects of growth, such as those involved in translation and cell division processes in *L. monocytogenes*, during growth in stationary phase, probably representing a physiological response to limiting nutrients. Regulation of these genes by σ^B ^has been observed before for other gram-positive bacteria [49]. The presence of a low number of consensus σ^B^-binding sequences (13) in down regulated genes as compared to 55 for σ^B ^up regulated genes indicates that repression by σ^B ^is often indirect and probably uses other signal transduction pathways and transcriptional regulators. Since detailed information about the role of σ^B ^as a negative regulator is scarce, further studies addressing this topic are needed.

### Orthologs of σ^B^-regulated genes absent in *L. innocua*

It was also of interest to determine how many of the σ^B ^regulon members of *L. monocytogenes *EGD-e were present/absent in the closely related non-pathogenic *L. innocua *as this is a free-living bacterium existing in the environment. Using the σ^B ^promoter consensus-directed search (GTTT-N_12–17_-GGGWAW), determined for *L. monocytogenes*, a majority of genes (~80%) were found in *L. innocua*, which were preceded by this sequence motif and consequently bear a significant homology to orthologs in *L. monocytogenes*. Thus the σ^B ^regulon appears to be highly conserved between these two listerial species.

Ten genes predicted to be involved in the stress response of *L. monocytogenes *had no orthologs in the non-pathogenic *L. innocua *strain. These include *lmo0819 *encoding a hypothetical protein as well as *lmo2387 *encoding a protein which is a member of a large and ubiquitously occurring family of voltage-gated sodium channels (CLC) in bacteria and vertebrates [50]. Other genes without a homolog in *L. innocua *were *lmo0263 *(Internalin H) and *lmo2085*, both encoding cell wall proteins with an LPXTG motif; *lmo0736*, which is similar to Ribose 5-phosphate isomerase; *lmo2157 *which encodes the previously described SepA required for septum formation, and *lmo2671 *encoding a lactoylglutathione lyase that catalyzes the initial step of the glyoxal pathway. Furthermore, two non-orthologous genes were identified as transcriptional regulator genes, *lmo0445 *and *lmo2672*. The gene product of *lmo0445 *harbors a Mga helix-turn-helix domain (Pfam prediction E-value 4.4 e^-29^). The second transcriptional regulator (*lmo2672*) belongs to the AraC family and could also be involved in acid response. In *E. coli*, low pH regulation is also dependent on two AraC-like regulators, GadW and GadX [51]. The gene products of *lmo0447 *and *lmo0448*, GadA and GadE, have been shown to be involved in acid resistance [52,53]. In our study, we confirmed that *lmo2067 *(bile salt hydrolase, *bsh*) [31,32], another *L. monocytogenes *specific gene locus, is regulated by σ^B^.

### Comparative genomic analysis of σ^B^-regulon members

The *sigB *operon in *L. monocytogenes *and *B. subtilis *are highly conserved with respect to structure and sequence similarity among the low G+C containing bacteria [16]. Additionally, the proposed σ^B^-binding consensus sequence is highly similar between these two species [23]. Of the 105 σ^B^-up regulated genes described in this study, we found 95 with orthologs in *L. innocua *and 75 with orthologs in *B. subtilis *through similarity searches (see Additional file 1, Table 1S). Of these 75 orthologous genes in *B. subtilis*, 33 have been previously reported to be σ^B^-dependent in *B. subtilis *[12,13]. These include well-characterised σ^B^-dependent genes such as, the members of the *sigB *operon itself and the osmoregulatory *opuC *operon (*opuCC*, *opuCB*), *csbD*, several universal/general stress genes and an arginine deiminase involved in acid tolerance [29]. This indicates conservation of a basic core set of stress responsive genes between these two species.

However, a larger set of genes are either disparately controlled between these two species or specific to *L. monocytogenes*. The former include genes encoding for transporters of di- and tripeptides, PTS systems, some genes involved in metabolism such as ribose-5-phosphate isomerase and acetate kinase as well as a large number of hitherto uncharacterized genes in *B. subtilis*. Examples of probable niche-specific σ^B^-regulated genes in *L. monocytogenes *include Internalin H, LPXTG- and LysM-motif containing cell wall proteins, putative collagen binding proteins and bile salt resistance and exclusion proteins [30,46]. In addition, many of the σ^B^-dependent effects are probably under the control of a secondary transcription regulatory circuit.

## Conclusion

We hypothesize that the current size of the σ^B ^regulon, comprising 216 up- and down-regulated genes and representing approx. 7.6% of all genes in the listerial genome. This compares with 128 genes or 3.1% of the genes for *B. subtilis *[13], 28 genes or 0.5% of the genes of *B. cereus *[17] and 251 genes or 9.6% of the genes of *S. aureus *[49], indicating that the alternative sigma factor directly regulates large sets of genes in these microorganisms. σ^B ^however must also exert its effect on many genes in an indirect manner, i.e. through control of secondary regulator genes and by inducing genes whose products stabilize RNAs posttranscriptionally, e.g through Hfq. Additional studies employing a variety of stress stimuli and growth conditions will be required to generate a comprehensive catalogue of σ^B^-regulated genes for *L. monocytogenes*.

## Methods

### Strains and growth conditions

*L. monocytogenes *EGD-e [34] and its isogenic deletion mutant Δ*sigB *(chromosomal deletion of region 930.725 bp – 931.393 bp) [54] and *S. aureus *COL [55] were used in this study. Bacteria were grown in brain heart infusion (BHI) broth (Difco) at 37°C with shaking (see Additional file 1, Fig. 4S) and where required supplemented with erythromycin at 5 μg/ml. *E. coli *InvαF' cells (Invitrogen) were grown in Luria-Bertani broth (LB) containing 300 μg/ml erythromycin to select for the plasmid pAUL-A. pSOG30222 was selected on BHI containing 5 μg/ml erythromycin.

### General molecular biological techniques

All oligonucleotide primers for PCR amplification used in this report for cloning procedures are listed in the supplementary material (see Additional file 1, Table 3S). Standard molecular techniques were used for DNA manipulation [56].

Plasmid DNA was transferred to *E. coli *InvαF' (Invitrogen) according to Hanahan [57]. The electroporation protocol of Park & Stewart [58] was used for the transformation of *L. monocytogenes *strains EGD-e and Δ*sigB*.

### Quantitative real-time PCR analysis

Quantitative real-time PCR was performed according to Chatterjee *et al. *[54]. Briefly, total RNA were isolated [54] from the wild type *L. monocytogenes *EGD-e growing in BHI medium for 3 h, 4 h, 8 h and 16 h (see Additional file 1, Fig. 4S). Following cDNA production the probes were subjected to quantitative real-time PCR in a final volume of 25 μl using RealMasterMix kit (Eppendorf) according to manufacturer's instruction. A standard curve was generated for *sigB *(*lmo0895*) primer pair (see Additional file 1, Table 3S) using known copy number of genomic DNA from *L. monocytogenes *EGD-e. For the primer pair a negative control (water), RNA sample without reverse transcriptase (to determine genomic DNA contamination) and sample with a known amount of template copies (to provide efficiency of the reaction) were included as controls during cDNA quantification. The experiment was carried out for three independent biological experiments.

### Production of σ^B ^specific anti-serum

To construct an *E. coli *vector expressing recombinant *S. aureus *σ^B ^appropriate DNA fragments were amplified using chromosomal DNA from *S. aureus *COL [55] and primers SigB_pPR_IBA1_for and SigB_pPR_IBA1_rev (see Additional file 1, Table 3S). The *Bsa*I digested DNA fragment was ligated into *Bsa*I digested pPRIBA-1 (IBA, Göttingen, Germany) to generate a Strep-tagged version of the protein.

For overexpression of recombinant σ^B ^the respective vector was transformed into *E. coli *BL21(DE3)/pLysS and a single colony propagated in one litre LB-medium until the culture reached an OD_540 _of 0.4. Synthesis of the protein was induced by the addition of IPTG (1 mM). Cells were harvested two hours after induction. Purification of proteins was performed according to the recommendations of the manufacturer (IBA, Göttingen, Germany). Purified σ^B ^was used for the immunisation of rabbits (Pineda, Berlin, Germany). The anti-σ^B ^serum was subjected to antigen specific affinity purification (Pane-Farre *et al*., manuscript in preparation).

### Preparation of protein extracts and immuno-blotting

Bacterial cultures in BHI were harvested after 3 h, 4 h, 8 h and 16 h of growth (see Additional file 1, Fig. 4S) by centrifugation at 5000 × *g *at 25°C for 10 min. Pellets were resuspended in PBS and cells were disrupted by using the Fastprep^®^-24 system (MP Biomedicals). Matrix B tubes were subjected to three 20 s bursts of agitation with breaks of 5 min on ice. Lysates were centrifuged at 13000 × *g *and supernatant containing released proteins was transferred to fresh tubes. The amount of protein in the various extracts was determined using a Bradford assay (Bio-Rad), and the fractions were stored at -20°C. Samples containing 12.5 μg proteins were separated by 12.5% SDS-PAGE, transferred to nitrocellulose membranes (Millipore) and probed with rabbit polyclonal anti-serum against *S. aureus *COL σ^B ^(Pane-Farre *et al*., manuscript in preparation). Detection was performed using horseradish peroxidase (Santa Cruz Biotechnology) -coupled anti-rabbit antibodies and the ECL kit (GE Health Care) as per the manufacturer's instructions.

### Transcriptome analysis

Microarray construction and expression profiling experiments were performed as previously described by Chatterjee *et al. *[54]. Briefly, overnight cultures of *L. monocytogenes *EGD-e cells (wild-type and mutant) were grown in brain heart infusion (BHI) broth (Difco). Bacteria were diluted 1:50 in 20 ml fresh BHI and incubated using a 100 ml Erlenmeyer flask at 37°C with shaking (180 rpm, Unitron [Infors]). 0.5 ml aliqouts of bacterial liquid cultures were taken at 3 h, 4 h, 8 h and 16 h of growth (see Additional file 1, Fig. 4S) and treated with 0.5 ml RNAprotect (Qiagen) according to manufacturer's protocol prior to centrifugation. The cells were frozen and stored at -80°C.

Total cellular RNA was extracted as previously described [54]. The quantity of the isolated total RNA was determined by absorbance at 260 nm and 280 nm, and the quality assessed using the Agilent 2100 Bioanalyzer. The isolated total RNA was stored at -80°C until used. cDNA was generated and labelled with CyDyes (both Cy3 and Cy5 for each probe) using 3 μg of total RNA with CyScribe Post-Labelling Kit (GE Health Care). The cDNA was quantified by absorbance at 550 nm and 650 nm for Cy3 and Cy5 respectively using ND-1000 Spectrophotometer (NanoDrop technologies, Inc., USA).

Hybridization was performed in ASP Base Hybridizer (GE Health Care) for 12 h at 42°C (as par Type 7* slide manufacturer's instruction) using 10 pmol of Cy dye incorporated cDNA of wild-type and mutant, 50 μl of hybridization buffer (GE Health Care) and 100 μl of deionized Formamide (Ambion) per slide. Hybridized Type 7*(star) slides (GE Health Care) were imaged with a Generation III Array Scanner (GE Health Care). The fluorescent signal intensities from each spot on the microarray were quantified using Spotfinder software (GE Health Care). Imaging and microarray analysis was performed as previously reported [54]. For each growth condition at least three independent biological RNA isolations were performed. Samples from each time-point were hybridized as dye swap experiments.

To identify differentially expressed genes between the wild-type and mutant strain, we used the Significance Analysis of Microarrays (SAM) program version 2.0 as described in the manual [59]. Briefly, SAM computes a statistic d_i _for each gene_i _measuring the strength of the relationship between gene expression and the response variable. It uses repeated permutations to determine whether the expression of any gene is significantly related to the response and creates a profile of observed versus expected values for each gene_i_. Genes with values outside a user-defined region of this profile are considered to be significantly regulated. Significance for each gene_i _is given as the false discovery rate (FDR). Here, data analysis was conducted with intra- and inter-slide normalized [54,60], log_2_-transformed data using the one class method in SAM 2.0 for the following comparisons of four different time points of growth: 1) *L. monocytogenes *EGD-e versus the deletion mutant Δ*sigB *at 3 h, 2) *L. monocytogenes *EGD-e versus the deletion mutant Δ*sigB *at 4 h, 3) *L. monocytogenes *EGD-e versus the deletion mutant Δ*sigB *at 8 h and 4) *L. monocytogenes *EGD-e versus the deletion mutant Δ*sigB *at 16 h. To identify σ^B^-dependent genes, a particular gene_i _was considered to be under σ^B ^control when (a) the FDR was < 1.0%, (b) the fold change 2-fold up or down regulated in the wild-type compared to the deletion mutant, and (c) when the gene was significantly regulated [see (a) and (b)] in at least two of the four growth conditions tested. Using these criteria, a final set of 216 significantly regulated genes was collated from all four experiments (see Additional file 1, Table 1S and Table 2S).

Microarray data have been deposited to ArrayExpress [61], accession number E-MEXP-1170.

Gene annotation was performed using the ERGO bioinformatics suite. To determine Clusters of Orthologous Groups of proteins (COGs) for *L. monocytogenes*, we used the organism-specific resource for microbial genomes provided by the NCBI webpage [62] Entrez Genomes. To investigate the specific role of each gene in metabolic pathways, the KEGG database for *L. monocytogenes *was used as a resource [63].

### Bioinformatic reevaluation of σ^B^-dependent genes

A consensus binding site for σ^B ^GTTT-N_13–17_-GGGWAW was previously reported based on Hidden Markov model (HMM)-based searches in the genome sequence of *L. monocytogenes *EGD-e [23]. We used the Gibbs sampling algorithm BIOPROSPECTOR [64] to search for motifs in the upstream region of the 216 genes identified above. We limited the search to 500 bp of the upstream sequence assuming that the σ^B^-binding sites should not be located further upstream than this distance from the gene under control. We identified the pattern GTTT-N_12–17_-GGGWAW as a reasonable σ^B^-binding motif. This pattern was used to perform an additional search using the tool fuzznuc from the EMBOSS package [65]. All sequences within 500 bp upstream of a putative σ^B^-dependent gene were tested for the motif, allowing one mismatch.

### Confirmation of positively σ^B^-regulated genes after transcriptome analysis by cloning σ^B^-dependent promoters

To investigate the role of σ^B ^as a transcriptional activator, we examined upstream regions of genes that were determined to be under positive control of σ^B ^from the microarray studies. The upstream regions of genes that were up regulated in the microarray experiments in *L. monocytogenes *EGD-e or down regulated in the isogenic deletion mutant Δ*sigB *were amplified by PCR using specific primers harboring *Bam*HI, *Pst*I or *Bgl*II restriction endonuclease cleavage sites (see Additional file 1, Table 3S, restriction endonuclease sites are underlined). The PCR fragments were cloned via a *Bam*HI/*Pst*I or *Bgl*II/*Pst*I restriction digestion into appropriate sites of the shuttle vector pSOG30222 (Fig. 1S, see supplementary material) [66] as a transcriptional fusion to the promotorless *bgaB *reporter gene and transformed into *E. coli *InvαF' electrocompetent cells. The insertion to the promotorless *bgaB *reporter gene was confirmed by sequencing, using primers pSOG30222_for _and pSOG30222_rev _(see Additional file 1, Table 3S). Recombinants were introduced both into the *L. monocytogenes *wild-type and its isogenic deletion mutant Δ*sigB*. The resulting strains were grown overnight (16 h) in brain heart infusion broth BHI (Difco) with erythromycin added to a final concentration of 5 μg/ml at 37°C, followed by measurement of the β-galactosidase activity. The vector without any promoter served as a negative control.

### Measurement of β-galactosidase activity of the σ^B^-dependent promoters

β-galactosidase assay experimental procedures were performed as originally described by Miller [67] and were optimised for the quantification of BgaB from *B. stearothermophilus *as reported by Schrogel & Allmannsberger [68]. Measurement of β-galactosidase activity was performed in triplicate using 500 μl of cell suspension and 300 μl of buffer B. The criterion for significant σ^B^-dependence of the promoters was set to at least 2-fold up regulation of the activity in the wild-type compared to its deletion mutant or *vice versa*.

### Comparative genomic analysis of σ^B^-regulon members

Homologs and orthologs in *B. subtilis *were determined by BLASTP results of the ProtTable for *L. monocytogenes *[69].

## Authors' contributions

TH and TC designed the research project. SH contributed to microarray probe design. TH constructed the *L. monocytogenes *EGD-e DNA microarray. SW provided the *sigB *deletion mutant. TH and SSC performed microarray experiments and TH, SSC, SO, UV and TC analyzed the microarray data. UV and SO provided the β-galactosidase gene reporter fusion experiments. JPF, SM and SE participated in the preparation of antibodies and immuno-blot analysis. BB and HH supported statistical evaluation of microarray data. AB and CTK were responsible for bioinformatic analysis of the experimental data. TH, HH, SSC, SO, UV and TC drafted the manuscript. All authors approved and read the final manuscript.

## Supplementary Material

Additional file 1 Supplementary material of temporal transcriptomic analysis of the *Listeria monocytogenes *EGD-e σ^B ^regulon. Material and methods, results and references for characterization of the chromosomal deletion mutant Δ*sigB *and conformation of bile susceptibility of the Δ*sigB *mutant. The file contains three tables and four figures: **Table 1S**. Overview of σB-dependent up regulated genes in *L. monocytogenes *EGD-e wild-type compared to the isogenic mutant Δ*sigB *from temporal transcriptomic analysis. **Table 2S**. Overview of σB-dependent down regulated genes in *L. monocytogenes *EGD-e wild-type compared to the isogenic mutant Δ*sigB *from temporal transcriptomic analysis. **Table 3S**. Primers used in this study. **Fig. 1S**. Survival of *L. monocytogenes *EGD-e wild type as compared to isogenic deletion mutant Δ*sigB *during growth in BHI at pH 7.0 and at low pH of 2.5. **Fig. 2S**. Map of the recombinant plasmid vector pSOG30222 used for the study of promoter activities in *L. monocytogenes *strains. **Fig. 3S**. Bile tolerance assay for *L. monocytogenes *EGD-e and Δ*sigB*. **Fig. 4S**. Growth at 37°C in BHI of *L. monocytogenes *EGD-e and Δ*sigB*. Click here for file
