# Supplementary material for: Temporal transcriptomic analysis of the Listeria monocytogenes EGD-e σB regulon
Source: BMC Microbiol. 2008 Jan 28;8:20. doi: 10.1186/1471-2180-8-20 (PMC2248587; doi:10.1186/1471-2180-8-20)

Supplementary material

Material and methods

Stress tolerance assays

To confirm the acid sensitive phenotype of the Δ*sigB* mutant and wild-type was compared at low pH. Wild-type and Δ*sigB* mutant cells were inoculated from an overnight BHI culture to 50 ml BHI medium in 1:50 dilution. At OD_{600} 0.4 the cells were divided into two 20 ml aliquots, harvested at 6000 × *g* for 15 min and pellets were re-suspended either in BHI (pH 7.0) or in BHI (pH 2.5) adjusted using HCl. Aliquots were taken every 10 min interval up to 30 min and plated on BHI agar plates in appropriate dilutions using the Autoplate 3000 spiral plating system (Spiral Biotech, USA). After 24 h of incubation at 37°C, the number of bacterial colonies was counted and total colony forming units per ml (CFU/ml) were determined.

Comparative bile tolerance of *L. monocytogenes* EGD-e and Δ*sigB* strains

Strains were screened for bile tolerance as described in [1] with few modifications. Briefly, overnight cultures were inoculated (3%) into BHI medium and into BHI medium containing 30% oxgall (B-3883; Sigma). The cultures were incubated for 30 min at 37°C without shaking using anaerobic jars and the Anaerocult A (Merck). Colony forming units (CFU) were measured by diluting cultures in one-quarter-strength PBS solution and enumeration on BHI-agar plates.

Results

Characterization of the chromosomal deletion mutant Δ*sigB*

We characterized the Δ*sigB* deletion mutant strain [2] used in this study as previously reported [3]. The survival rate of Δ*sigB* mutant was determined at highly acidic (pH 2.5) and standard BHI medium (pH 7.0) and compared to that of the wild-type at 10 min intervals for up to 30 min (Fig. 1S). The survival rate of the Δ*sigB* mutant was reduced significantly compared to the wild-type strain in BHI at low pH already following incubation for 10 minutes. After 20 min of incubation, no mutant bacteria survived at low pH. Growth of either bacteria in BHI broth at pH of 7.0 revealed no differences in colony forming units (CFU) numbers for both strains. Thus, the Δ*sigB* deletion mutant was more susceptible to exposure to low pH as compared to the wild-type strain.

Conformation of bile susceptibility of the Δ*sigB* mutant

The microarray analysis confirmed σ^B-dependent regulation of the bile salt hydrolase (*bsh, lmo2067*) [4] and the bile exclusion system (*bileEAB*, *lmo1421-22*) [5], both being essential factors required for survival in the infected host. We examined the survival ability of *L. monocytogenes* EGD-e and its isogenic Δ*sigB* mutant in the presence of growth media BHI and BHI containing 30% oxgall. The cultures were anaerobically incubated at 37°C and plated on BHI-agar plates for CFU counts. Our results indicated (Fig. 3S) the decrease in the number of bacteria grown without 30% oxgall (BHI [t=30min]). The Δ*sigB* strain grown in BHI containing 30% oxgall is highly sensitive to bile challenge as compared to the wild-type (BHI+30%Oxgall [t=30min]) as previously reported [6].

References

1. Begley M, Gahan CG, Hill C: **Bile stress response in *Listeria monocytogenes* LO28: adaptation, cross-protection, and identification of genetic loci involved in bile resistance.** *Appl Environ Microbiol* 2002, **68:** 6005-6012.
2. Chatterjee SS, Hossain H, Otten S, Kuenne C, Kuchmina K, Machata S, Domann E, Chakraborty T, Hain T: **Intracellular gene expression profile of *Listeria monocytogenes*.** *Infect Immun* 2006, **74:** 1323-1338.
3. Ferreira A, Sue D, O'Byrne CP, Boor KJ: **Role of *Listeria monocytogenes* σ^B in survival of lethal acidic conditions and in the acquired acid tolerance response.** *Appl Environ Microbiol* 2003, **69:** 2692-2698.
4. Sue D, Boor KJ, Wiedmann M: **σ^B-dependent expression patterns of compatible solute transporter genes *opuCA* and *lmo1421* and the conjugated bile salt hydrolase gene *bsh* in *Listeria monocytogenes*.** *Microbiology* 2003, **149:** 3247-3256.
5. Sleator RD, Wemekamp-Kamphuis HH, Gahan CG, Abee T, Hill C: **A PrfA-regulated bile exclusion system (BilE) is a novel virulence factor in *Listeria monocytogenes*.** *Mol Microbiol* 2005, **55:** 1183-1195.
6. Begley M, Sleator RD, Gahan CG, Hill C: **Contribution of three bile-associated loci, *bsh, pva,* and *btlB,* to gastrointestinal persistence and bile tolerance of *Listeria monocytogenes*.** *Infect Immun* 2005, **73:** 894-904.

Figure legends

Fig. 1S Survival of *L. monocytogenes* EGD-e wild type as compared to isogenic deletion mutant Δ*sigB* during growth in BHI at pH 7.0 [EGD-e (filled circles); Δ*sigB* (filled triangles)] and at low pH of 2.5 [EGD-e (open circles); Δ*sigB* (open triangles)]. After overnight incubation, strains were cultured (1:50) up to OD_{600} 0.4. Bacterial cells were divided into two aliquots, harvested by centrifugation and re-suspended either in BHI (pH 7.0) or in BHI (pH 2.5). Incubation of bacteria were carried out up to 30 min and plated on agar plates for CFU counts. The experiment was replicated thrice. An asterisk indicates that the means are significantly different from the wild type during growth in BHI at low pH of 2.5 ($P < 0.0067$).

Fig. 2S Map of the recombinant plasmid vector pSOG30222 used for the study of promoter activities in *L. monocytogenes* strains. Features of relevance to the study are indicated in the figure. *bgaB* w/o P = promoterless β-galactosidase from *B. stearothermophilus*; *cat* w/o P = promoterless chloramphenicol acetyltransferase gene from plasmid pTV53; *repR*=replication initiator protein from pIP501, *ermC*= erythromycin resistance gene from pE194; ColE1= ColE1 replicon. Restriction endonuclease cloning sites: *Sma*I, *Pst*I, *Eco*RI, *Xho*I, *Bgl*II and *Spe*I.

Fig. 3S Bile tolerance assay for *L. monocytogenes* EGD-e and Δ*sigB*. Strains were exposed to 30% oxgall under anaerobic conditions. CFU counts of bacteria growing in BHI under anaerobic condition was measured at time=0 [BHI(t=0)] and at time=30 min in the absence [BHI(t=30)] and presence of 30% oxgall [BHI+30%Oxgall(t=30)] (black bars: EGD-e; shaded bars: Δ*sigB*). The experiment was replicated thrice. An asterisk indicates that the means are significantly different from the wild type ($P < 0.037$).

Fig. 4S Growth at 37°C in BHI of *L. monocytogenes* EGD-e (squares, full line) and Δ*sigB* (circles, dashed line). The data are the averages of three independent growth experiments.

Table 1S. Overview of σ^B-dependent up regulated genes in *L. monocytogenes* EGD-e wild-type compared to the isogenic mutant Δ*sigB* from temporal transcriptomic analysis

Gene	Strand	Annotation	3h-fold change	4h-fold change	8h-fold change	16h-fold change	*in vivo* system *L. monocytogenes*	σ^B consensus box	σ^B box position	Hom. in *L. innocua*	Hom. in *B. subtilis*	Hom. σ^B reg. in *B. subtilis*	COG no. and description
lmo0019	-	Hypothetical protein	**4.2**	**10.0**	**2.5**	**2.0**		CTTTTTATTTTTCCAAAATAGGGTAT	-57	*lin0018*	*yvpB*		COG4990 Uncharacterized protein conserved in bacteria
lmo0043	+	Arginine deiminase	**5.4**	**5.8**	**2.2**	**2.4**	2.5±1.0	GCTTTTTTTTATTTCCCGGGAAA	-72	*lin0036*	*ykgA*	+[a,b]	COG2235 Arginine deiminase
lmo0134	+	Acetyltransferase	**2.3**	**2.2**	**4.1**	2.8				*lin0181*			COG2388 Predicted acetyltransferase
lmo0169	+	Glucose uptake protein	**4.0**	**3.7**	**2.1**	**2.4**	**21.1±0.9**	GATTTCATGAGGAAAAGGGTAT	-56	*lin0212*	*ycxE*		COG4975 Putative glucose uptake permease
lmo0170	+	Phosphatidylserine decarboxylase	**3.9**	**2.5**	**2.1**	**2.7**				*lin0213*			COG5361 Uncharacterized conserved protein
lmo0263[c]	+	Internalin H	2.0	**3.0**	2.1	**3.5**		GTTAATTTGGTCTAAAAAAGGGTAT	-90				No COG
lmo0265	+	Succinyl-diaminopimelate desuccinylase	**3.3**	**3.5**	**9.5**	**8.5**	**120.0±1.9**	GTTTGCCTTTATAGAGAACGGGAAA	-61	*lin0289*	*argE*		COG0624 Acetylornithine deacetylase/Succinyl-diaminopimelate desuccinylase and related deacylases
lmo0405[c]	+	Low-affinity inorganic phosphate transporter	**2.0**	1.5	**2.5**	**2.5**	**83.9±7.6**	TTTTATATTTGTATAAAAGGGTAT	-65	*lin0428*	*ykaB*		COG0306 Phosphate/sulphate permeases
lmo0439	-	Surfactin synthetase related protein	**4.9**	**3.0**	**2.2**	**2.8**	**50.4±10.2**	GTTTCACAACACTGCTTTCAGGGAAA	-66	*lin0460*			COG4908 Uncharacterized protein containing a NRPS condensation (elongation) domain
lmo0445	+	Trans-acting positive regulator	**5.5**	**5.7**	**2.2**	**2.7**	**16.8±1.2**				*licR*	+[b]	No COG
lmo0515	+	Universal stress protein	1.5	1.4	**2.9**	**3.5**				*lin0515*	*nhaX*	+[a]	COG0598 Universal stress protein UspA and related nucleotide-binding proteins
lmo0529	+	N-acetylglucosaminyltransferase	n.d.	n.d.	**2.1**	**3.0**				*lin0533*	*ydaM*		COG1215 Glycosyltransferases, probably involved in cell wall biogenesis
lmo0539	-	Tagatose-bisphosphate aldolase	**12.0**	**10.3**	**5.4**	**7.9**	**23.2±1.3**	GTTTTAAAAAAATTATTCAGTGGTAT	-87	*lin0543*			COG3684 Tagatose-1,6-bisphosphate aldolase
lmo0554	+	NADH-dependent butanol dehydrogenase	**6.3**	**3.9**	**2.3**	1.9	**135.6±19.6**	GTTTAAATTTTCTAAAAAAGTGTAT	-73	*lin0563*	*yugJ*	+[a]	COG1454 Alcohol dehydrogenase, class IV
lmo0555	+	Di-/tripeptide transporter	**3.7**	**2.8**	1.9	1.9				*lin0564*	*yclF*		COG3104 Dipeptide/tripeptide permease
lmo0580	+	Carboxylesterase	**2.5**	1.8	1.9	**2.3**				*lin0589*	*yodD*		COG0400 Predicted esterase
lmo0590	+	Predicted kinase related to hydroxyacetone kinase	**2.4**	**2.2**	1.7	2.0				*lin0599*	*yloV*		COG1461 Predicted kinase related to dihydroxyacetone kinase
lmo0591	+	Membrane spanning protein	**2.2**	**2.1**	1.2	1.1				*lin0600*			COG3752 Predicted membrane protein
lmo0593[c]	+	Formate/Nitrite transporter	1.7	**5.4**	**2.1**	2.3		GTTTTAAGAGTTTGAAAACGGGAAA	-182	*lin0602*	*yrhG*		COG2116 Formate/nitrite family of transporters
lmo0596	+	Hypothetical protein	**40.5**	**32.6**	**7.9**	**6.5**	**2.3±0.2**	GTTTTAAATTCGTTTTTTAGGCTAT	-99	*lin0605*			COG3247 Uncharacterized conserved protein
lmo0602	+	Acetyltransferase	**3.5**	**3.9**	2.1	**3.6**	**78.7±0.7**	GTTTCATCAATTTGCGCAAGTGAAA	-37	*lin0611*	*yuaI*	+[a]	COG0454 Histone acetyltransferase HPA2 and related acetyltransferases
lmo0610	-	Internalin protein	**2.8**	1.8	**2.1**	**2.4**	**169.0±8.3**	GTTTAACATATTACTAAAAGAGGAAT	-84	*lin0619*			No COG
lmo0647	-	Hypothetical protein	**3.0**	**2.0**	**4.9**	**8.6**				*lin0650*			No COG
lmo0669[c]	+	Glucose-1-dehydrogenase	**7.1**	**17.0**	**2.2**	**3.3**	**54.2±27.9**	GTTTTAGCGTAAAACAGGAGGGAAG	-174	*lin0674*	*yhxD*	+[b]	COG1028 Dehydrogenases with different specificities (related to short-chain alcohol dehydrogenases)
lmo0670	+	Hypothetical protein	**3.9**	**9.2**	2.1	**3.0**				*lin0675*	*ydaT*	+[a,b]	COG4876 Uncharacterized protein conserved in bacteria
lmo0676	+	Flagellar biosynthesic protein FliP	**5.5**	**4.7**	1.7	1.5	**2.1±0.1**			*lin0684*	*fliP*		COG1338 Flagellar biosynthesis pathway, component FliP
lmo0722	+	Pyruvate oxidase	**5.7**	**5.3**	**2.2**	**2.8**	**18.5±1.1**			*lin0730*	*ydaP*	+[a,b]	COG0028 Thiamine pyrophosphate-requiring enzymes
lmo0735	+	Ribulose-phosphate 3-epimerase	n.d.	1.1	**3.4**	**4.7**				*lin2811*	*yloR*		COG0036 Pentose-5-phosphate-3-epimerase
lmo0736	+	Ribose 5-phosphate isomerase	n.d.	n.d.	**4.9**	**5.8**					*ywlF*		COG0698 Ribose 5-phosphate isomerase RpiB
lmo0737	+	Hypothetical protein	n.d.	n.d.	**3.3**	**4.3**				*lin0026*			No COG
lmo0738	+	PTS system, beta-glucoside-specific IIABC component	n.d.	n.d.	**3.2**	**3.6**				*lin0288*	*glvC*		COG1264 Phosphotransferase system IIB components
lmo0739	+	6-phospho-beta-glucosidase	1.2	1.1	**2.3**	**2.9**				*lin0742*	*bglH*		COG2723 Beta-glucosidase/6-phospho-beta-glucosidase/beta-galactosidase
lmo0781	-	PTS system, mannose-specific IID component	**7.7**	**9.5**	**2.2**	**3.9**				*lin0774*	*levG*		COG3716 Phosphotransferase system, mannose/fructose/N-acetylgalactosamine-specific component IID
lmo0782	-	PTS system, mannose-specific IIC component	**9.4**	**10.6**	**2.3**	**4.3**				*lin0775*	*levF*		COG3715 Phosphotransferase system, mannose/fructose/N-acetylgalactosamine-specific component IIC
lmo0783	-	PTS system, mannose-specific IIAB component	**10.9**	**11.4**	**2.2**	**4.3**	**67.2±25.1**			*lin0776*	*levE*		COG3444 Phosphotransferase system, mannose/fructose/N-acetylgalactosamine-specific component IIB
lmo0784[c]	-	PTS system, mannose-specific IIA component	1.4	n.d.	**2.1**	**2.7**		GTTTTCTGACTAATCTTTTAGGGTAA	-231	*lin0777*	*levD*		COG2893 Phosphotransferase system, mannose/fructose-specific component IIA
lmo0794[c]	-	Putative NAD-dependent dehydrogenase	**5.9**	**8.4**	**3.5**	**6.6**	**61.4±2.5**	GTTTCCCAGTCCCCTCTTTCGGGAAT	-81	*lin0787*	*ywnB*		COG2910 Putative NADH-flavin reductase
lmo0796	-	Hypothetical protein	**9.8**	**6.9**	**2.5**	**4.4**	**9.3±0.2**			*lin0789*			COG2353 Uncharacterized conserved protein
lmo0819	+	Hypothetical protein	1.5	1.4	**2.1**	**2.6**							No COG
lmo0869	+	Hypothetical protein	1.3	1.1	**2.1**	**2.2**				*lin0862*			COG2159 Predicted metal-dependent hydrolase of the TIM-barrel fold
lmo0880[c]	+	Collagen adhesion protein	**6.6**	**11.2**	**2.9**	**5.9**	**100.5±6.0**	GTTTTTAACAAGCAAGTTGTGGGAAC	-62	*lin0879*	*yojL*		COG1388 FOG: LysM repeat
lmo0895[c]	+	RNA polymerase sigma B	**13.0**	**20.3**	1.8	2.4				*lin0894*	*sigB*	+[a,b]	COG1191 DNA-directed RNA polymerase specialized sigma subunit
lmo0911[c]	+	Hypothetical protein	**5.2**	**4.0**	**2.7**	**3.9**	**228.3±4.6**	GTTTTAACTTGCCCTCAGGCGGGTAT	-89	*lin0911*			COG3708 Uncharacterized protein conserved in bacteria
lmo0913	+	Succinate-semialdehyde dehydrogenase [NADP+]	**9.2**	**21.6**	**3.7**	**8.5**	**163.8±26.3**			*lin0913*	*ycnH*	+[b]	COG1012 NAD-dependent aldehyde dehydrogenases
lmo0953	+	Hypothetical protein	**6.4**	**14.1**	**3.6**	**3.5**		GTTTTACTTCTACTTTTTTAGGGAAT	-58	*lin0952*			No COG
lmo0956[c]	+	N-acetylglucosamine-6-phosphate deacetylase NagA	**2.2**	1.7	**2.1**	**2.5**		GGTTATTTTACTTTTTTTCGGGTAA	-106	*lin0955*	*nagA*		COG1820 N-acetylglucosamine-6-phosphate deacetylase
lmo0957	+	Glucosamine-6-phosphate isomerase NagB	1.7	1.6	**2.0**	**2.4**				*lin0956*	*nagB*		COG0363 6-phosphogluconolactonase/Glucosamine-6-phosphate isomerase/deaminase
lmo0994[c]	-	Hypothetical protein	**9.5**	**13.0**	**3.7**	**5.8**	**165.7±14.4**	GTTTAGCCGCTTAACAAAACGGGAAA	-63	*lin0993*			No COG
lmo0997	-	ClpE	n.d.	1.7	**3.5**	**7.4**				*lin0996*	*clpE*		COG0542 ATPases with chaperone activity, ATP-binding subunit
lmo1055	+	E3 subunit of pyruvate dehydrogenase complex PdhD	1.1	**2.2**	n.d.	**2.2**				*lin1047*	*acoL*		COG1249 Pyruvate/2-oxoglutarate dehydrogenase complex, dihydrolipoamide dehydrogenase (E3) component
lmo1068	+	Hypothetical protein	1.2	n.d.	**2.8**	**3.3**				*lin1056*			No COG
lmo1140	-	Hypothetical cytosolic protein	**2.8**	**2.6**	**3.0**	**5.4**				*lin1104*			COG3865 Uncharacterized protein conserved in bacteria
lmo1168	+	Acetate kinase	**4.0**	**3.9**	1.7	1.8		GTTTACTCCTTTAAAAATGAGAAA	-36	*lin1132*	*ackA*		COG0282 Acetate kinase
lmo1241	+	CDP-4-dehydro-6-deoxy-D-glucose 3-dehydratase	**3.4**	**3.2**	**2.2**	**2.7**	**4.7±0.3**	GATTGAGCATCCAAAAACAGGGGTAT	-70	*lin1205*			COG5361 Uncharacterized conserved protein
lmo1261	-	Membrane spanning protein	**3.8**	**2.3**	1.6	1.5		GTTTAACTTTTAGCGTTTTTGGGAAT	-68	*lin1229*	*ypdC*		COG2339 Predicted membrane protein
lmo1295	+	Hfq	**2.2**	**2.2**	2.9	1.4		GTTTGGTAAGAAGAAATAAAGGGTAT	-113	*lin1333*	*ymaH*		COG1923 Uncharacterized host factor I protein
lmo1375	+	Peptidase T	**3.0**	**4.4**	1.8	1.8		GTTTTAATCTAGGTTTAGCGGGTAT	-367	*lin1412*	*yqjE*		COG2195 Di- and tripeptidases
lmo1421[c]	+	Glycine betaine/L-proline transport ATP-binding protein	**3.4**	**3.8**	1.6	1.2		GTTTTTTTCTTAAATTAGGAAT	-128	*lin1460*	*opuCA*	+[a]	COG1125 ABC-type proline/glycine betaine transport systems, ATPase components
lmo1422	+	Glycine betaine transport system permease and binding proteir	**2.7**	**4.7**	1.4	1.4				*lin1461*	*opuCB*	+[a]	COG1174 ABC-type proline/glycine betaine transport systems, permease component
lmo1425[c]	-	Glycine betaine/L-proline transport system permease protein	**8.8**	**6.3**	1.6	2.0				*lin1464*	*opuCD*	+[a]	COG1174 ABC-type proline/glycine betaine transport systems, permease component
lmo1426[c]	-	Glycine betaine-binding protein	**10.6**	**8.6**	1.7	2.3				*lin1465*	*opuCC*	+[a]	COG1732 Periplasmic glycine betaine/choline-binding (lipo)protein of an ABC-type transport system
lmo1427[c]	-	Glycine betaine transport system permease protein	**5.7**	**9.9**	1.4	1.7				*lin1466*	*opuCB*	+[a]	COG1174 ABC-type proline/glycine betaine transport systems, permease component
lmo1428[c]	-	Glycine betaine/L-proline transport ATP-binding protein	**4.9**	**4.4**	1.5	1.6	**24.2±4.0**	GTTTAAATCTATACTAGTTAGGGAAA	-84	*lin1467*	*opuCA*	+[a]	COG1125 ABC-type proline/glycine betaine transport systems, ATPase components
lmo1433[c]	-	Glutathione reductase	**2.5**	**3.3**	**2.2**	**2.7**	2.0±0.1	GTTTGAAAGTGAAATCAGACGGGAAA	-50	*lin1472*	*pdhD*		COG1249 Pyruvate/2-oxoglutarate dehydrogenase complex, dihydrolipoamide dehydrogenase (E3) component
lmo1526	-	Hypothetical protein	**3.3**	**2.4**	**2.2**	**2.9**	**222.7±16.3**	GTTTTTAATAGGACAGAAACGGGTAC	-58	*lin1561*	*yrvD*		No COG
lmo1580[c]	+	Universal stress protein	**2.5**	1.3	**2.8**	**3.6**		GGTTCTTTTAGGAAAAAGAGGGTAA	-70	*lin1615*	*yxiE*	+[a]	COG0589 Universal stress protein UspA and related nucleotide-binding proteins
lmo1601	-	General stress protein	**2.5**	1.6	**2.7**	2.2				*lin1642*	*ytxH*	+[a,b]	COG4980 Gas vesicle protein
lmo1602[c]	-	General stress protein	**2.7**	1.9	**3.2**	**3.1**	**3.3±0.4**	GTTTTAGAGGGGAATACTCAGGGTAT	-54	*lin1643*	*ytxG*	+[a]	COG4768 Uncharacterized protein containing a divergent version of the methyl-accepting chemotaxis-like domain
lmo1606[c]	-	Cell division protein FtsK	**2.3**	**3.1**	1.8	1.9		GTTTAACCCCTCTATTACCAAGGTAT	-133	*lin1647*	*ytpT*		COG1647 DNA segregation ATPase FtsK/SpoIIIE and related proteins
lmo1637	+	Bacitracin transport permease protein BcrB	**5.6**	**6.9**	**3.6**	**4.7**	**5.8±0.6**			*lin1678*	*yhcI*	+[a]	COG1277 ABC-type transport system involved in multi-copper enzyme maturation, permease component
lmo1694[c]	+	Cell division inhibitor	**6.4**	**6.1**	**4.5**	**5.0**	**21.6±5.9**	GTTTTAATACTACTAAAAAGGGAAT	-57	*lin1802*	*yfhF*	+[a,b]	COG1090 Predicted nucleoside-diphosphate sugar epimerase
lmo1830	+	Short chain dehydrogenase	**4.6**	**11.0**	**3.5**	**4.5**	**169.1±29.6**	GTTTTTTCTTTCTAATTTTAGGGTAG	-69	*lin1944*	*ykvO*	+[a,b]	COG1028 Dehydrogenases with different specificities (related to short-chain alcohol dehydrogenases)
lmo1883[c]		Chitinase	1.3	**3.0**	**4.1**	**6.8**		GTTTTATTTTCACTATGTTGGGTAT	-82	*lin1996*			No COG
lmo2067[c]	-	Bsh	**3.2**	**3.0**	**3.0**	2.4	1.1±0.1	GTTTTACTCCAAACTCCGAGGGTAC	-66		*yxeI*		COG3049 Penicillin V acylase and related amidases R51
lmo2085[c]	-	Collagen adhesion protein	**3.3**	**15.5**	**4.7**	**9.4**	**199.6±34.8**	GTTTTCTTTTGCTGTTTTATGGGTAT	-64				COG4932 Predicted outer membrane protein
lmo2157[c]	-	Alkyl sulfatase SepA	**6.6**	**13.0**	2.2	1.3		GTTTTGAATAATTTTATGGAGGTAT	-69				COG2015 Alkyl sulfatase and related hydrolases
lmo2158	-	CsbD	**12.9**	**10.6**	**26.1**	**10.4**		GTTTTAGCTTTCTATATTGTGGAAA	-86	*lin2261*	*ywmG*	+[a]	COG3237 Uncharacterized protein conserved in bacteria
lmo2175	-	Short chain dehydrogenase	n.d.	n.d.	**2.1**	**3.6**		GATTATAATAAAAATAGAAAGGGAAT	-69	*lin2278*	*dhbA*		COG1028 Dehydrogenases with different specificities (related to short-chain alcohol dehydrogenases)
lmo2205[c]	-	Phosphoglycerate mutase	**3.1**	**2.2**	**3.4**	**5.7**		GTTTGACACTTCACTTGAAAGGGAAA	-80	*lin2308*	*yhfR*		COG0588 Phosphoglycerate mutase 1
lmo2230	+	Arsenate reductase	**39.2**	**158.0**	**11.5**	**21.5**		GTTTCTAGTAATTTAAAAAGGGTAG	-143	*lin2332*	*yqcM*	+[a,b]	COG0394 Protein-tyrosine-phosphatase
lmo2231	+	Cobalt-zinc-cadmium resistance protein CzcD	**4.3**	**6.5**	**2.4**	1.5	2.5±0.3			*lin2333*	*ydbO*		COG0053 Predicted Co/Zn/Cd cation transporters
lmo2386[c]	+	Hypothetical membrane spanning protein	**2.3**	**2.6**	1.8	1.5		GTTTTTAATAAGCTCATTGTGGTAAA	-58	*lin2485*	*yuiD*		COG2485 Uncharacterized protein conserved in bacteria
lmo2387	+	Chloride channel	**2.9**	**2.9**	**3.5**	**3.1**	**224.9±5.7**	GTTTACAGCTATATCTTAAAGGGAAA	-65				COG0038 Chloride channel protein EriC
lmo2391	+	Putative NAD-dependent dehydrogenase	**9.8**	**9.1**	**11.2**	**16.2**	**9.4±1.8**	GTTTTATTTTTTACTCACCGGGAAA	-61	*lin2490*	*yhfK*	+[a]	COG0702 Predicted nucleoside-diphosphate-sugar epimerases
lmo2398[c]	+	Low temperature requirement C protein	**3.4**	**3.1**	1.5	1.1				*lin2497*	*yutG*	+[a]	COG1267 Phosphatidylglycerophosphatase A and related proteins
lmo2463[c]	-	Transporter, Mmpl family	**3.9**	**9.7**	2.3	**2.6**		GTTTGGCATATGTAAAAAAGAGGTAT	-34	*lin2557*	*ydfJ*		COG2409 Predicted drug exporters of the RND superfamily
lmo2484	-	Integral membran protein	**3.8**	**4.9**	**2.2**	2.7				*lin2627*	*yvlD*	+[a]	COG1950 Predicted membrane protein
lmo2485[c]	-	Stress-responsive transcriptional regulator PspC	**3.0**	**3.5**	2.2	2.4	1.8±0.1	GTTTAATAAAATGAAAGGAAGGGAAA	-29	*lin2628*	*yvlC*	+[a]	COG1983 Putative stress-responsive transcriptional regulator
lmo2494	-	regulator of phosphate regulon PhoU	1.7	**2.6**	**2.4**	1.8				*lin2637*			COG0704 Phosphate uptake regulato
lmo2511[c]	-	Sigma 54 modulation protein/ribosomal protein S30EA	1.9	n.d.	**4.7**	**4.7**		GTTTTCACGTAGACTAAGGGTAA	+76	*lin2655*	*yvyD*	+[a]	COG1544 Ribosome-associated protein Y (PSrp-1)
lmo2570[c]	-	Hypothetical protein	**4.4**	**6.0**	**3.1**	**5.0**				*lin2715*	*yvaZ*		No COG
lmo2571	-	Pyrazinamidase/Nicotinamidase	**4.0**	**7.1**	**3.6**	**5.9**				*lin2716*	*yueJ*	+[b]	COG1335 Amidases related to nicotinamidase
lmo2572	-	Dihydrofolate/Pyrimidine reductase family protein	**3.8**	**6.1**	**3.1**	**5.2**				*lin2717*	*ywjB*		COG0262 Dihydrofolate reductase
lmo2573	-	NADPH:quinone reductase	**7.5**	**11.6**	**3.5**	**5.7**	**82.7±6.1**	GTTTTAAAGCCTTCTCAGTGAAT	-96	*lin2718*	*yogA*		COG0604 NADPH:quinone reductase and related Zn-dependent oxidoreductases
lmo2602[c]	+	Membrane protein, MgtC/SapB family	**4.4**	**8.7**	**3.8**	**7.1**	**82.1±12.6**	GTTTTGGTTTAATGCCAAAGGGAAT	-51	*lin2751*	*sapB*		COG1285 Uncharacterized membrane protein
lmo2603	+	Acetamidase	**3.2**	**7.5**	**3.6**	**4.8**				*lin2572*			COG2421 Predicted acetamidase/formamidase
lmo2671	-	Lactoylglutathione lyase	**2.6**	**2.6**	**3.2**	2.3							No COG
lmo2672	-	Transcriptional regulator, AraC family	**2.3**	**3.2**	**2.9**	**3.1**					*ybfI*	+[a]	COG2207 AraC-type DNA-binding domain-containing proteins
lmo2673[c]	+	Universal stress protein	**6.9**	**16.7**	**50.6**	**49.5**	**87.9±4.0**	GTATTAAGTAAATAAGGGGAAA	-44	*lin2820*	*yxiE*	+[a]	COG0589 Universal stress protein UspA and related nucleotide-binding proteins
lmo2674	+	Ribose-5-phosphate isomerase	**2.5**	1.9	**13.4**	**11.9**				*lin2821*	*ywlF*		COG0698 Ribose 5-phosphate isomerase RpiB
lmo2695[c]	+	Dihydroxyacetone kinase	**10.9**	**14.9**	**5.0**	**24.6**	**10.3±0.5**	GTTTTGACTTTCTAGTAAAGGGAAA	-65	*lin2843*			COG2376 Dihydroxyacetone kinase
lmo2696	+	Dihydroxyacetone kinase	**10.6**	**4.8**	**6.0**	**20.5**				*lin2844*			COG2376 Dihydroxyacetone kinase
lmo2697	+	Dihydroxyacetone kinase phosphotransfer protein	**14.9**	**12.6**	**6.2**	**23.2**				*lin2845*			COG3412 Uncharacterized protein conserved in bacteria
lmo2724	-	PhnB protein	**4.9**	**5.0**	1.3	1.3	**45.5±2.3**	GTTTAAGGTAAAACGAATTGGGTAT	-63	*lin2872*			COG2764 Uncharacterized protein conserved in bacteria
lmo2748	-	General stress protein 26	**10.8**	**22.1**	**5.5**	**8.0**	**47.2±3.5**	GTTTGAAGCCGGGAGACGAGTGGAAA	-59	*lin2891*	*ydaG*	+[a,b]	COG3871 Uncharacterized stress protein (general stress protein 26)

Putative operons are boxed; annotation of genes corresponds to ERGO bioinformatics suite; significant fold change (in bold) indicates the ratio of expression intensities of the wild-type compared to its Δ*sigB* strain; the abbreviation n.d.=not detected is included for gene expression values that were not expressed under the conditions used; *in vivo* system indicates the results from the reporter gene assay (significant values are marked in bold, $P < 0.00023$); σ^B consensus box indicates the presence of a putative σ^B-binding sequence (in bold) using Bioprospector [64] and the position of the σ^B consensus box upstream of the start codon, mismatches are underlined, homologs and orthologs in *B. subtilis* were determined by BLASTP results of the ProtTable for *L. monocytogenes* [69] and annotated as σ^B regulated following ([a]) Price *et al.*, 2001 and ([b]) Petersohn *et al.*, 2001; ([c]) indicates σ^B-up regulated genes of *L. monocytogenes* strain 10403S identified by Kazmierczak *et al.*, 2003; Functional categories were determined using COG's for *L. monocytogenes* provided by NCBI [62].

Table 2S. Overview of σ^B-dependent down regulated genes in *L. monocytogenes* EGD-e wild-type compared to the isogenic mutant Δ*sigB* from temporal transcriptomic analysis

Gene	Strand	Annotation	8h-fold change	16h-fold change	σ^B consensus box	σ^B box position	*Hom. in L. innocua*	COG no. and description
lmo0190	+	4-Diphosphocytidyl-2-C-methyl-D-erythritol kinase	6.0	3.6			*lin0229*	COG1947 4-diphosphocytidyl-2C-methyl-D-erythritol 2-phosphate synthase
lmo0218	+	Polyribonucleotide nucleotidyltransferase domain protein	4.8	3.6			*lin0250*	COG1185 Polyribonucleotide nucleotidyltransferase (polynucleotide phosphorylase)
lmo0220	+	Cell division protein FtsH	2.9	2.6			*lin0252*	COG0465 ATP-dependent Zn proteases
lmo0228	+	Lysyl-tRNA synthetase LysL	4.3	2.7			*lin0260*	COG1190 Lysyl-tRNA synthetase (class II)
lmo0582	-	Iap, P60	6.1	4.9	**GTTT**TTTAGGTGAAAACT**GGGTAA**	-110	*lin0591*	No COG
lmo0663	-	Conserved hypothetical protein	4.0	3.1			*lin0668*	COG0561 Predicted hydrolases of the HAD superfamily
lmo0811	+	Carbonic anhydrase	3.1	3.1			*lin0807*	COG3338 Carbonic anhydrase
lmo0970	+	Enoyl-acyl-carrier protein reductase	2.6	2.4			*lin0969*	COG0623 Enoyl-[acyl-carrier-protein] reductase (NADH)
lmo0993	+	Potassium uptake protein	4.6	2.4			*lin0992*	COG0168 Trk-type K+ transport systems, membrane components
lmo1071	+	Cell division protein FtsW	2.3	2.1			*lin1059*	COG0772 Bacterial cell division membrane protein
lmo1086	+	D-Ribitol-5-phosphate cytidylyltransferase	2.6	3.2			*lin1071*	COG1211 4-diphosphocytidyl-2-methyl-D-erithritol synthase
lmo1087	+	Ribitol-5-phosphate 2-dehydrogenase	2.1	2.2			*lin1072*	COG1063 Threonine dehydrogenase and related Zn-dependent dehydrogenases
lmo1287	+	Topoisomerase IV subunit A	2.4	2.2			*lin1326*	COG0188 Type IIA topoisomerase (DNA gyrase/topo II, topoisomerase IV), A subunit
lmo1314	+	Ribosome recycling factor, Frr	2.4	2.4			*lin1351*	COG0233 Ribosome recycling factor
lmo1351	-	Rhodanese-related sulfurtransferase	3.1	2.1	**GTTT**TCAGGATGAAAG**GGGTCT**	-28	*lin1388*	COG0607 Rhodanese-related sulfurtransferase
lmo1358	+	Hypothetical cytosolic protein	4.2	4.0			*lin1395*	COG1302 Uncharacterized protein conserved in bacteria
lmo1359	+	N utilization substance protein B	2.4	2.0			*lin1396*	COG0781 Transcription termination factor
lmo1365	+	1-Deoxy-D-xylulose 5-phosphate synthase	2.0	2.0			*lin1402*	COG1154 Deoxyxylulose-5-phosphate synthase
lmo1424	-	Manganese transport protein MntH	6.0	3.9			*lin1463*	COG1914 Mn2+ and Fe2+ transporters of the NRAMP family
lmo1431	+	ABC transporter ATP-binding protein	2.7	2.4	**GTTT**TGTGCTTTTTTC**GGCAAT**	-496	*lin1470*	COG0488 ATPase components of ABC transporters with duplicated ATPase domains
lmo1440	+	Hypothetical protein	2.5	2.4			*lin1479*	No COG
lmo1450	-	ATP-dependent RNA helicase	4.8	4.1			*lin1488*	COG0513 Superfamily II DNA and RNA helicases
lmo1456	-	Hypothetical protein	2.5	2.0			*lin1493*	COG1671 Uncharacterized protein conserved in bacteria
lmo1480	+	Ribosomal protein S20 RpsT	2.2	2.8			*lin1515*	COG0268 Ribosomal protein S20
lmo1488	-	Nicotinate-nucleotide adenylyltransferase	2.6	2.3			*lin1523*	COG1057 Nicotinic acid mononucleotide adenylyltransferase
lmo1496	-	Transcription elongation factor GreA	2.0	2.0			*lin1531*	COG0782 Transcription elongation factor
lmo1504	-	Alanyl-tRNA synthetase AlaS	2.7	3.1	**GTTT**TCCCATGGCTGGA**AGGAAA**	-213	*lin1539*	COG0013 Alanyl-tRNA synthetase
lmo1520	-	Histidyl-tRNA synthetase HisS	11.6	4.2	**GTTT**TTATAACAAGTGACGG**GGGTTT**	-64	*lin1555*	COG0124 Histidyl-tRNA synthetase
lmo1540	-	Ribosomal protein L27 RpmA	2.5	2.6			*lin1575*	COG0211 Ribosomal protein L27
lmo1542	-	Ribosomal protein L21 RplU	2.8	2.5			*lin1577*	COG0261 Ribosomal protein L21
lmo1544	-	Cell division inhibitor MinD	2.8	2.1			*lin1579*	COG2894 Septum formation inhibitor-activating ATPase
lmo1546	-	Rod shape-determining protein MreD	2.6	2.0			*lin1581*	No COG
lmo1574	-	DNA polymerase III alpha subunit	2.3	2.1			*lin1609*	COG0587 DNA polymerase III, alpha subunit
lmo1581	-	Acetate kinase	3.5	2.0			*lin1616*	COG0282 Acetate kinase
lmo1585	-	Putative signal peptide peptidase	2.4	2.0			*lin1627*	COG0616 Periplasmic serine proteases (ClpP class)
lmo1596	+	Ribosomal protein S4 RpsD	3.0	2.5			*lin1638*	COG0522 Ribosomal protein S4 and related proteins
lmo1647	+	1-Acyl-sn-glycerol-3-phosphate acyltransferase	2.2	2.3			*lin1688*	COG0204 1-acyl-sn-glycerol-3-phosphate acyltransferase
lmo1657	-	Protein translation elongation factor Tsf	6.1	3.3	**TTTT**AAAGAGAAAAATTGGA**GGGAAA**	-30	*lin1766*	COG0264 Translation elongation factor Ts
lmo1663	-	Asparagine synthetase (glutamine-hydrolyzing)	2.4	2.3			*lin1772*	COG0367 Asparagine synthase (glutamine-hydrolyzing)
lmo1670	+	Hypothetical cytosolic protein	3.0	2.3			*lin1778*	COG0759 Uncharacterized conserved protein
lmo1676	-	Isochorismate synthase	2.4	2.3	**GTCT**ACCCTTCATTTTA**GGGAAA**	-137	*lin1784*	COG1169 Isochorismate synthase
lmo1677	+	1,4-Dihydroxy-2-naphthoate octaprenyltransferase	6.7	4.5			*lin1785*	COG1575 1,4-dihydroxy-2-naphthoate octaprenyltransferase
lmo1681	-	5-Methyltetrahydropteroyltriglutamate/Homocysteine methyltransferase	2.5	2.6			*lin1789*	COG0620 Methionine synthase II (cobalamin-independent)
lmo1724	-	ABC transporter ATP-binding protein	3.2	2.3			*lin1835*	COG1131 ABC-type multidrug transport system, ATPase component
lmo1746	-	ABC transporter permease protein	7.2	4.4			*lin1857*	COG0577 ABC-type antimicrobial peptide transport system, permease component
lmo1752	-	Hypothetical protein	3.2	2.7			*lin1864*	No COG
lmo1776	-	Hypothetical protein	2.8	2.5			*lin1888*	No COG
lmo1785	-	Bacterial protein translation initiation factor InfC	5.5	3.9			*lin1897*	COG0290 Translation initiation factor 3 (IF-3)
lmo1804	-	Chromosome partition protein Smc	10.0	4.3			*lin1918*	COG1196 Chromosome segregation ATPases
lmo1805	-	Ribonuclease III	2.6	2.2			*lin1919*	COG0571 dsRNA-specific ribonuclease
lmo1808	-	Malonyl-CoAacyl-carrier-protein transacylase	2.2	2.2			*lin1922*	COG0331 (acyl-carrier-protein) S-malonyltransferase
lmo1814	-	Predicted kinase related to hydroxyacetone kinase	2.4	3.0			*lin1928*	COG1461 Predicted kinase related to dihydroxyacetone kinase
lmo1818	-	Ribulose-phosphate 3-epimerase	2.6	3.2			*lin1932*	COG0036 Pentose-5-phosphate-3-epimerase
lmo1831	-	Orotate phosphoribosyltransferase	2.2	2.1			*lin1945*	COG0461 Orotate phosphoribosyltransferase
lmo1847	-	Manganese-binding protein MntA	10.0	5.0			*lin1961*	COG0803 ABC-type metal ion transport system, periplasmic component/surface adhesin
lmo1848	-	Manganese transport system membrane protein MntC	10.8	6.1			*lin1962*	COG1108 ABC-type Mn2+/Zn2+ transport systems, permease components
lmo1849	-	Manganese transport system ATP-binding protein MntB	6.3	3.2	**GTTT**TGCCGTTTTTTCT**GAGTAT**	-239	*lin1963*	COG1121 ABC-type Mn/Zn transport systems, ATPase component
lmo1875	-	ABC transporter ATP-binding protein	5.6	3.6			*lin1989*	COG0488 ATPase components of ABC transporters with duplicated ATPase domains
lmo1937	-	GTP-binding protein	5.6	3.4	**GTTT**GATAAATAGGATTC**GAGAAA**	-39	*lin2051*	COG1160 Predicted GTPases
lmo2032	-	Cell division protein FtsZ	3.3	3.1			*lin2138*	COG0206 Cell division GTPase
lmo2034	-	Cell division protein FtsQ	5.3	4.5			*lin2140*	COG1589 Cell division septal protein
lmo2039	-	Division specific D,D-transpeptidase/Cell division protein FtsI	2.8	2.5			*lin2145*	COG0768 Cell division protein FtsI/penicillin-binding protein 2
lmo2040	-	Cell division protein FtsL	2.2	3.4			*lin2146*	COG4839 Protein required for the initiation of cell division
lmo2048	-	Hypothetical cytosolic protein	6.0	4.1	**GTTT**GTGCCCATTATGAAAA**GGGTTA**	-184	*lin2154*	COG1399 Predicted metal-binding, possibly nucleic acid-binding protein
lmo2062	-	Copper resistance protein	2.9	2.5			*lin2168*	COG1276 Putative copper export protein
lmo2192	-	Oligopeptide transport ATP-binding protein OppF	2.1	2.1			*lin2296*	COG4608 ABC-type oligopeptide transport system, ATPase component
lmo2194	-	Oligopeptide transport system permease protein OppC	3.1	2.8			*lin2298*	COG1173 ABC-type dipeptide/oligopeptide/nickel transport systems, permease components
lmo2201	-	3-Oxoacyl-acyl-carrier-protein synthase	2.6	3.5			*lin2304*	COG0304 3-oxoacyl-(acyl-carrier-protein) synthase
lmo2244	+	Ribosomal large subunit pseudouridine synthase D	3.1	3.2			*lin2346*	COG0564 Pseudouridylate synthases, 23S RNA-specific
lmo2245	-	Hypothetical protein	4.4	2.5			*lin2347*	COG0346 Lactoylglutathione lyase and related lyases
lmo2254	+	Guanine-hypoxanthine permease	2.3	2.8			*lin2356*	COG2252 Permeases
lmo2334	-	Transcriptional regulator, MerR family	2.9	2.7			*lin2428*	COG1396 Predicted transcriptional regulators
lmo2367	-	Glucose-6-phosphate isomerase	3.5	4.2	**TTTT**TTAAAATTTGGA**GGGAAA**	-25	*lin2466*	COG0166 Glucose-6-phosphate isomerase
lmo2371	+	ABC transporter permease protein	2.4	2.7			*lin2470*	COG0577 ABC-type antimicrobial peptide transport system, permease component
lmo2415	-	ABC transporter ATP-binding protein	2.9	2.4			*lin2510*	COG0396 ABC-type transport system involved in Fe-S cluster assembly, ATPase component
lmo2429	-	Ferrichrome ABC transporter: ATP-binding protein FhuC	7.6	4.6			*lin2523*	COG1120 ABC-type cobalamin/Fe3+-siderophores transport systems, ATPase components
lmo2449	-	Exoribonuclease II	4.6	2.9			*lin2543*	COG0557 Exoribonuclease R
lmo2450	-	Carboxylesterase	2.9	2.7			*lin2544*	COG0596 Predicted hydrolases or acyltransferases (alpha/beta hydrolase superfamily)
lmo2451	-	Protein translocase subunit SecG	3.8	2.4			*lin2545*	COG1314 Preprotein translocase subunit SecG
lmo2456	-	Phosphoglycerate mutase	2.1	2.1			*lin2550*	COG0696 Phosphoglyceromutase
lmo2457	-	Triosephosphate isomerase	2.3	2.1			*lin2551*	COG0149 Triosephosphate isomerase
lmo2475	-	Phosphoglucomutase/Phosphomannomutase	4.5	5.8			*lin2618*	COG1109 Phosphomannomutase
lmo2486	-	Hypothetical protein	2.8	2.8			*lin2629*	COG1983 Putative stress-responsive transcriptional regulator
lmo2503	-	Cardiolipin synthetase	4.2	3.1	**GTTT**GTGTAAGAAAAGG**GGGAAC**	-26	*lin2646*	COG1502 Phosphatidylserine/phosphatidylglycerophosphate/cardiolipin synthases and related enzymes
lmo2505	-	N-acetylmuramoyl-L-alanine amidase Spl/C120P45	4.1	5.0			*lin2649*	No COG
lmo2506	-	Cell division protein FtsX	4.7	5.7			*lin2650*	COG2177 Cell division protein
lmo2507	-	Cell division ATP-binding protein FtsE	2.0	5.7			*lin2651*	COG2884 Predicted ATPase involved in cell division
lmo2508	-	Conserved hypothetical proteins	2.2	2.1			*lin2652*	COG1284 Uncharacterized conserved protein
lmo2510	-	Protein translocase subunit SecA	6.5	5.1			*lin2654*	COG0653 Preprotein translocase subunit SecA (ATPase, RNA helicase)
lmo2542	-	Methyltransferase	5.2	3.9			*lin2686*	COG2890 Methylase of polypeptide chain release factors
lmo2547	-	Homoserine dehydrogenase	2.4	2.5			*lin2691*	COG0460 Homoserine dehydrogenase
lmo2553	-	Conserved hypothetical protein	2.7	2.4			*lin2698*	COG0392 Predicted integral membrane protein
lmo2554	-	Glycosyltransferase	3.2	2.9			*lin2699*	COG0438 Glycosyltransferase
lmo2560	-	DNA-directed RNA polymerase delta chain RpoE	2.6	2.6			*lin2705*	COG3343 DNA-directed RNA polymerase, delta subunit
lmo2562	-	Hypothetical protein	3.0	4.6			*lin2707*	No COG
lmo2596	-	Ribosomal protein S9 RpsI	2.5	2.1			*lin2745*	COG0103 Ribosomal protein S9
lmo2597	-	Ribosomal protein L13 RplM	8.7	3.8	**GTTT**CAATTTATTTAGGA**GGGTAA**	-27	*lin2746*	COG0102 Ribosomal protein L13
lmo2618	-	Ribosomal protein S8 RpsH	2.6	2.0			*lin2767*	COG0096 Ribosomal protein S8
lmo2622	-	Ribosomal protein L14 RplN	2.7	2.2			*lin2771*	COG0093 Ribosomal protein L14
lmo2626	-	Ribosomal protein S3 RpsC	2.2	2.1			*lin2775*	COG0092 Ribosomal protein S3
lmo2627	-	Ribosomal protein L22 RplV	2.7	2.7			*lin2776*	COG0091 Ribosomal protein L22
lmo2628	-	Ribosomal protein S19 RpsS	2.4	2.2			*lin2777*	COG0185 Ribosomal protein S19
lmo2629	-	Ribosomal protein L2 RplB	2.6	2.2			*lin2778*	COG0090 Ribosomal protein L2
lmo2631	-	Ribosomal protein L4 RplD	3.9	3.5			*lin2780*	COG0088 Ribosomal protein L4
lmo2634	-	Cobalt transport protein CbiQ	2.4	2.0			*lin2783*	COG0619 ABC-type cobalt transport system, permease component CbiQ and related transporters
lmo2641	+	Farnesyl pyrophosphate synthetase/Geranyltranstransferase	2.8	2.3			*lin2790*	COG0142 Geranylgeranyl pyrophosphate synthase
lmo2710	-	Hypothetical protein	3.3	2.8			*lin2858*	No COG
lmo2712	-	Gluconokinase	2.2	2.0			*lin2860*	COG1070 Sugar (pentulose and hexulose) kinases
lmo2758	-	Inosine-5-monophosphate dehydrogenase	2.0	2.4			*lin2901*	COG0516 IMP dehydrogenase/GMP reductase
lmo2777	+	Bicyclomycin resistance protein	2.1	2.4			*lin2916*	COG0477 Permeases of the major facilitator superfamily
lmo2785	-	Catalase	4.1	2.9			*lin2920*	COG0753 Catalase

Putative operons are boxed; annotation of genes corresponds to ERGO bioinformatics suite; significant fold change (in bold) indicates the ratio of expression intensities of the wild-type compared to its Δ*sigB* strain; σ^B consensus box indicates the presence of a putative σ^B-binding sequence (in bold) using Bioprospector [64] and the position of the σ^B consensus box upstream of the start codon, mismatches are underlined; functional categories were determined using COG's for *L. monocytogenes* provided by NCBI [62].

Table 3S. Primers used in this study

Primer	Sequence (5`- 3`)	Reference
lmo0043$_{\text{p-for}}$	AAGGATCCTTGCTAGATTCATGCC	This work
lmo0043$_{\text{p-rev}}$	ATCACCTCTGCAGAACTAATTACC	This work
lmo0169$_{\text{p-for}}$	TTCTCCGGATCCATCACAAACCAAGC	This work
lmo0169$_{\text{p-rev}}$	ATTCTGCAGCTTATGGTCTATACC	This work
lmo0265$_{\text{p-for}}$	TGTGGATCCAAATGACAATAATTCAG	This work
lmo0265$_{\text{p-rev}}$	TTCCTGCAGTCTTTCTATGAATTCCAC	This work
lmo0405$_{\text{p-for}}$	ATGCGGATCCACGCGGAATGAATGC	This work
lmo0405$_{\text{p-rev}}$	AGCAGTCTGCAGTGTCGAGATTCAC	This work
lmo0439$_{\text{p-for}}$	TAGTAAGCGGATCCTTTCCATC	This work
lmo0439$_{\text{p-rev}}$	ATGAAAAAGCCTGCAGTCTTCGG	This work
lmo0445$_{\text{p-for}}$	AAGGATCCTCTTGAGGGAGTGTC	This work
lmo0445$_{\text{p-rev}}$	ATATCCTGCAGTACAAATGACC	This work
lmo0539$_{\text{p-for}}$	TCCTTTACTGCAGTTTGTTGTTGC	This work
lmo0539$_{\text{p-rev}}$	CATGGATCCTGGTCTAGTCTTATCG	This work
lmo0554$_{\text{p-for}}$	CTCTTGGATCCTTGCATCTCCAGG	This work
lmo0554$_{\text{p-rev}}$	TCTTTCTGTTTCTGCAGTTACATAGC	This work
lmo0596$_{\text{p-for}}$	ATCTTTCAGGATCCACTGCTGG	This work
lmo0596$_{\text{p-rev}}$	ACTCTGCAGTATTTTTTCTAATTCAACC	This work
lmo0602$_{\text{p-for}}$	GATGGATCCTTTACCAAACAGGTG	This work
lmo0602$_{\text{p-rev}}$	ATCATCCATTCTGCAGTCACTTGC	This work
lmo0610$_{\text{p-for}}$	TGCAGGATCCAAAGAAGCAGTAG	This work
lmo0610$_{\text{p-rev}}$	TTTCTGCAGTATAATCGTATGGATTC	This work
lmo0669$_{\text{p-for}}$	CCATTCTGGATCCATTTAGTGTCGTTC	This work
lmo0669$_{\text{p-rev}}$	TTGCTGCAGAACCCATGACTAAGT	This work
lmo0676$_{\text{p-for}}$	TTTAGGGATCCAATCACATACCTC	This work
lmo0676$_{\text{p-rev}}$	TTCCATTCACTGCAGGTAAATTCACC	This work
lmo0722$_{\text{p-for}}$	GTCGAAGGATCCTTGGCAGAACG	This work
lmo0722$_{\text{p-rev}}$	TCATTTTACTTTCTGCAGCCATGGATG	This work
lmo0783$_{\text{p-for}}$	TACGGATCCTTGAATGAAATCCAACC	This work
lmo0783$_{\text{p-rev}}$	TAGGTATCTCCTGCAGAAGCGTGG	This work
lmo0794$_{\text{p-for}}$	ACCAATGGATCCATGCAGTACG	This work
lmo0794$_{\text{p-rev}}$	AAACTGCAGCCTATTCATTTTTATGG	This work
lmo0796$_{\text{p-for}}$	CTTGGATCCTATTCTACGCAC	This work
lmo0796$_{\text{p-rev}}$	AGCATTCTCTGCAGATCTAGC	This work
lmo0880$_{\text{p-for}}$	AAGTGGATCCTCGCGCTATCATTG	This work
lmo0880$_{\text{p-rev}}$	TTACTGCAGCTATCTTTATAGTTCC	This work
lmo0911$_{\text{p-for}}$	TTGGTTGGAGGATCCATACAGC	This work
lmo0911$_{\text{p-rev}}$	ACTCTTCTGCAGAAGCATCTCAC	This work
lmo0913$_{\text{p-for}}$	TTTGGGATCCTATTCTACTTCCTTGC	This work
lmo0913$_{\text{p-rev}}$	TCTGCAGATTTTTCTGCACGTGCTCTCG	This work
lmo0994$_{\text{p-for}}$	TATGGATCCTTTGTGGAAGTTAAGTCG	This work
lmo0994$_{\text{p-rev}}$	ATACAACCTGCAGTTTCTATCTATACG	This work
lmo1241$_{\text{p-for}}$	AGGGATCCAAGTTCGATTCATGG	This work
lmo1241$_{\text{p-rev}}$	TTCATGTAAAACTGCAGCTTCAATTAGC	This work
lmo1428$_{\text{p-for}}$	CGGGATCCTATTTTTGGCACTATGTTG	This work
lmo1428$_{\text{p-rev}}$	ATCCTGCAGTTTTTCTTTACCACTACCA	This work

Restriction endonuclease sites are underlined.

Primer	Sequence (5`- 3`)	Reference
lmo1433$_{p-for}$	ATCAGGATCCATTACTTCAGGACC	This work
lmo1433$_{p-rev}$	AATCACTGCAGCTTAGCTTGTTTTCC	This work
lmo1526$_{p-for}$	TAGTGGATCCATTCTGAACTTCTCC	This work
lmo1526$_{p-rev}$	TCCCTGCAGGATTATCTTATTCTG	This work
lmo1602$_{p-for}$	TGCATCTCTCATATTGGATCCTC	This work
lmo1602$_{p-rev}$	ATCTGCAGTATACCCTGAG	This work
lmo1637$_{p-for}$	TACGGATCCAGGCGAAACAGAC	This work
lmo1637$_{p-rev}$	AACTCACTCCTGCAGTTCTCTCAC	This work
lmo1694$_{p-for}$	CGGGATCCTTTCGAACCATATCAGCTTG	This work
lmo1694$_{p-rev}$	ATCCTGCAGGTCCTAACTAGTTTATTCC	This work
lmo1830$_{p-for}$	AAGGGATCCTTTCAGCAACAGTCC	This work
lmo1830$_{p-rev}$	TACTGCAGTAAAGTCAGTACACATC	This work
lmo2067$_{p-for}$	CTCTGGATCCTCTACCTTGCG	This work
lmo2067$_{p-rev}$	AAGAGGTCTGCAGACGAGTGTAC	This work
lmo2085$_{p-for}$	TTTGGATCCACAGGATGAGCAAGTAAG	This work
lmo2085$_{p-rev}$	ATACCTTCTGCAGCCTTTTCCCTG	This work
lmo2231$_{p-for}$	TTAGGATCCTAGTTCAGAATTACTGGC	This work
lmo2231$_{p-rev}$	TACTGCAGTCTCTTTTAACATAAAAGTCC	This work
lmo2387$_{p-for}$	AACGGGATCCCAAGACTTCGTGG	This work
lmo2387$_{p-rev}$	CTTCTGCAGATCCGTTGATATCATG	This work
lmo2391$_{p-for}$	CGGGATCCGAAACGCTAGAGATTGACG	This work
lmo2391$_{p-rev}$	ATCCTGCAGAAAAATATCTACAAAGAAC	This work
lmo2485$_{p-for}$	TAGAGGATCCAAATGGTG	This work
lmo2485$_{p-rev}$	TTTTCACTGCAGTCCCTTCCTTTC	This work
lmo2573$_{p-for}$	TATCGGGATCCTAGCAATGTTCAC	This work
lmo2573$_{p-rev}$	AACACCTGCAGTTTCCTTTTCCCG	This work
lmo2602$_{p-for}$	AACCATTGGATCCAACAAGTGCAACC	This work
lmo2602$_{p-rev}$	TTACTGCAGGTTAATATATTCCC	This work
lmo2673$_{p-for}$	AAGGATCCATCACTAACGCCAAC	This work
lmo2673$_{p-rev}$	TTGCTGCAGCCTCTCAAAAGTTTTCC	This work
lmo2695$_{p-for}$	AATGGATCCACTTTTGTGTCCAG	This work
lmo2695$_{p-rev}$	AACCTGCAGTATAATAGTCCTCTTACC	This work
lmo2724$_{p-for}$	TTGGATCCATGCTTCTAGCTGCTCC	This work
lmo2724$_{p-rev}$	AATCGCTGCAGTGTTTTTGAGACAC	This work
lmo2748$_{p-for}$	AAACTGGATCCAGAGACAACC	This work
lmo2748$_{p-rev}$	ACTGCAGTTTGGTTATTTAGTGTACC	This work
pSOG30222$_{for}$	CGATTTTTGTGATGCTCGTCAG	This work
pSOG30222$_{rev}$	CCATCAGACGGTTCGATCTTG	This work
Lmo0895_5	CTGTAGATCATTCGATTGAAGC	This work
Lmo0895_3	TCTTCTGTTCTCGCTCATCTAA	This work
SigB_pPR_IBA1_for	ATGGTAGGTCTCAAATGGCGAAAGAGTCGAAATCAGCTAAT	This work
SigB_pPR_IBA1_rev	ATGGTAGGTCTCAGCGCTTTGATGTGCTGCTTCTTGTAATTTC	This work

Restriction endonuclease sites are underlined.

Fig. 1S

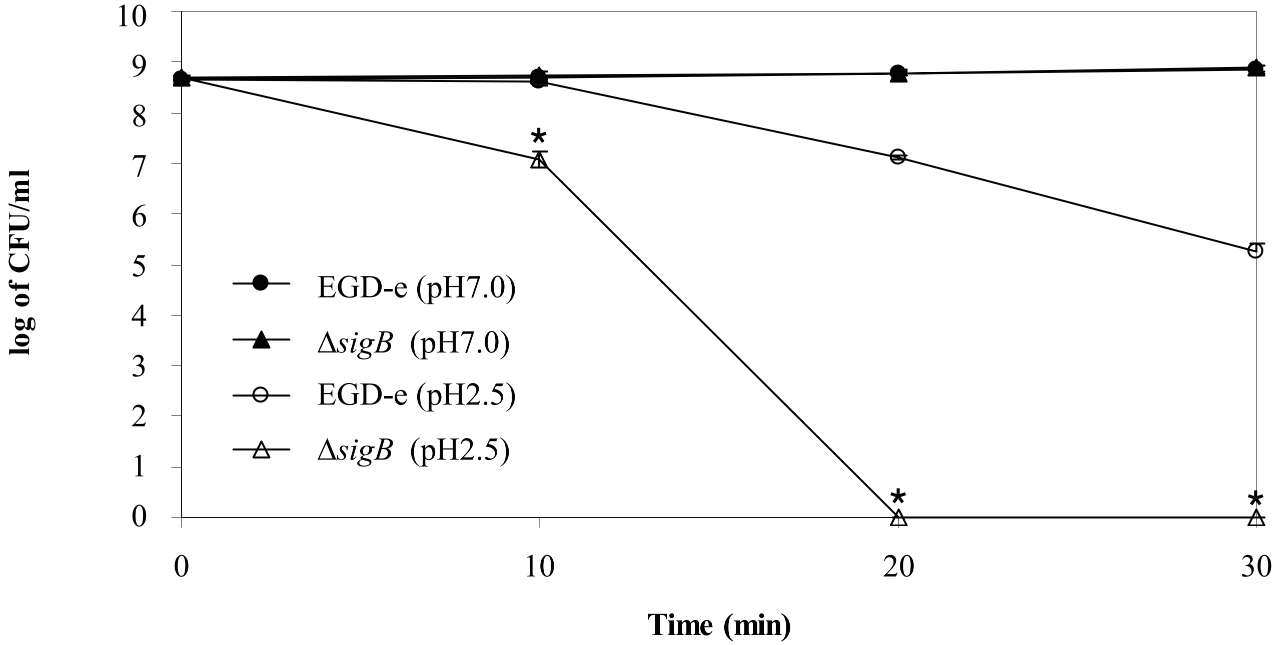

10
9
8
7
6
5
4
3
2
1
0
log of CFU/ml
*
*
*
EGD-e (pH7.0)
ΔsigB (pH7.0)
EGD-e (pH2.5)
ΔsigB (pH2.5)
0
10
20
30
Time (min)

Fig. 2S

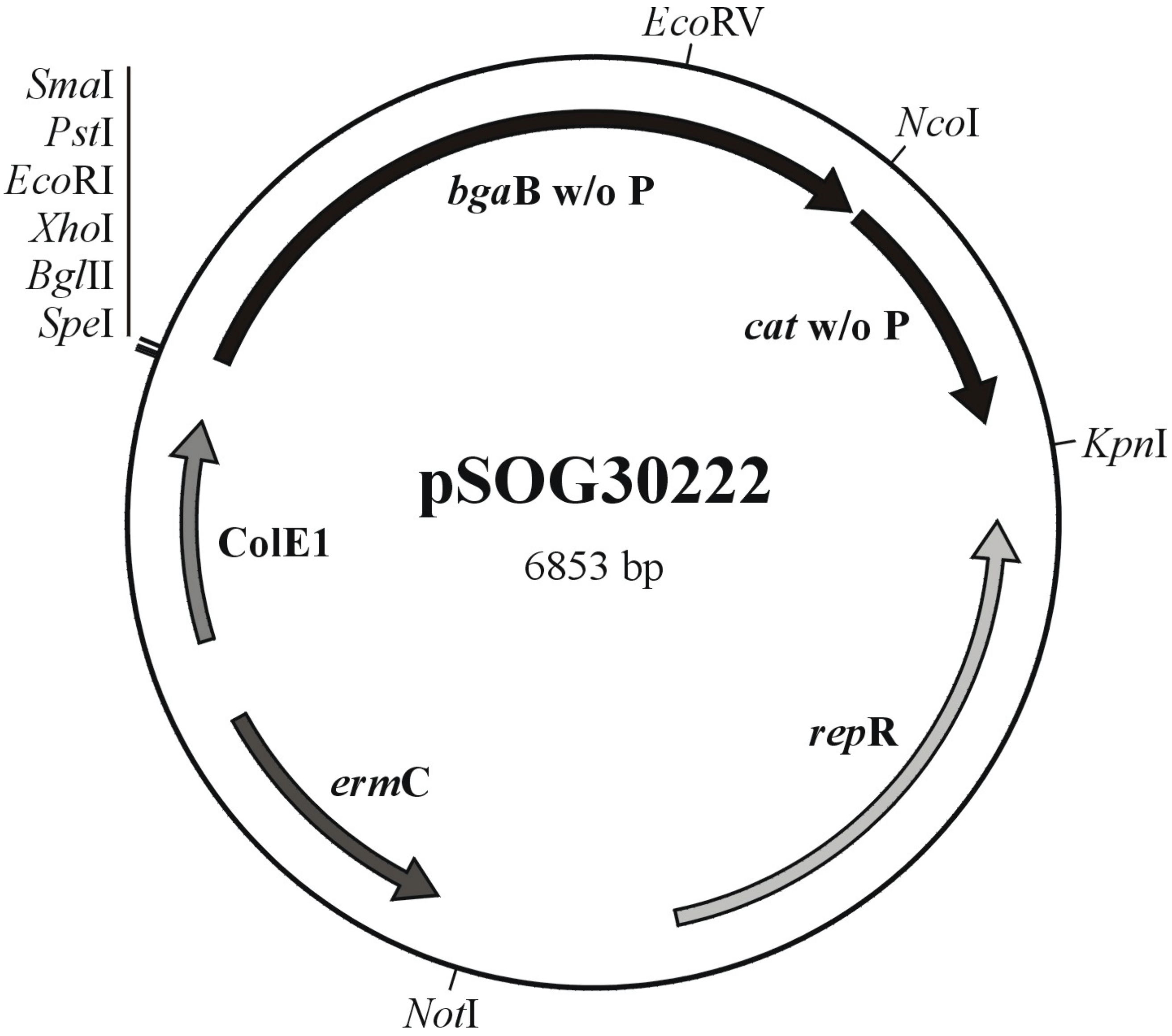
*Eco*RV
*Sma*I
*Pst*I
*Eco*RI
*Xho*I
*Bgl*II
*Spe*I
*Nco*I
***bga*B w/o P**
***cat* w/o P**
*Kpn*I
pSOG30222
6853 bp
ColE1
***erm*C**
***rep*R**
*Not*I

Fig. 3S

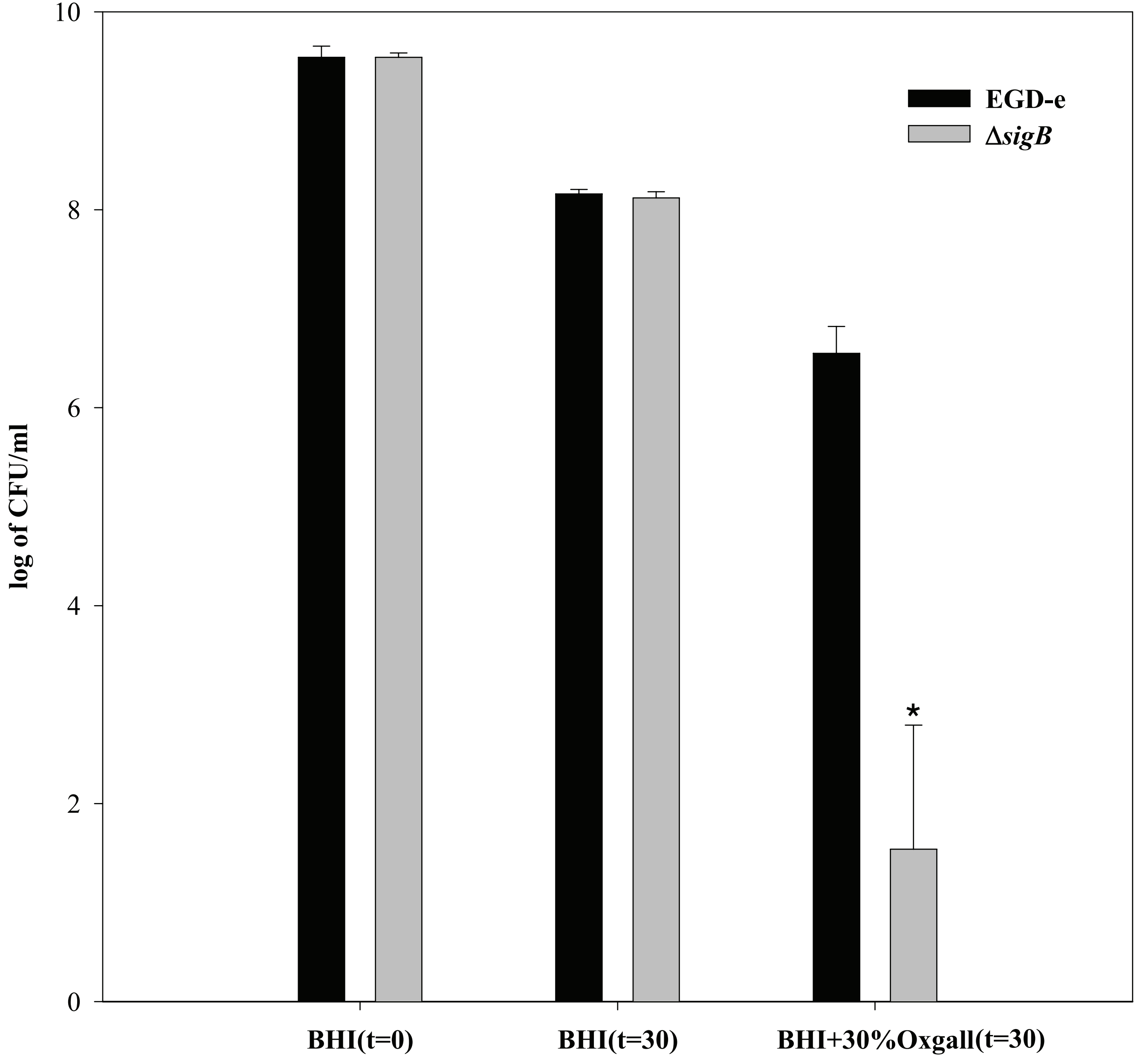
EGD-e
ΔsigB
log of CFU/ml
0
2
4
6
8
10
*
BHI(t=0)
BHI(t=30)
BHI+30%Oxgall(t=30)

Fig. 4S

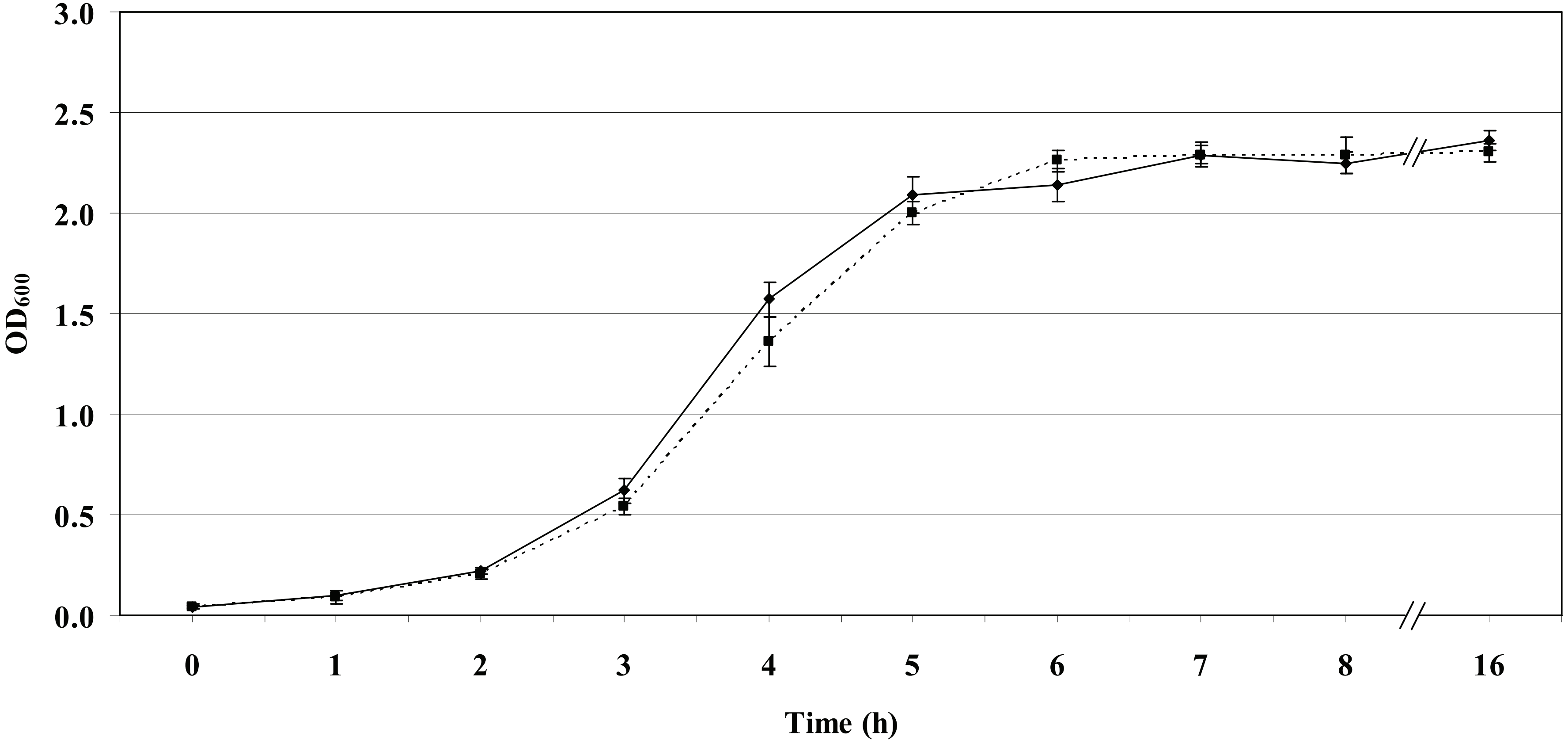

Supplement: Additional file 1 — Supplementary material of temporal transcriptomic analysis of the Listeria monocytogenes EGD-e σB regulon. Material and methods, results and references for characterization of the chromosomal deletion mutant ΔsigB and conformation of bile susceptibility of the ΔsigB mutant. The file contains three tables and four figures: Table 1S. Overview of σB-dependent up regulated genes in L. monocytogenes EGD-e wild-type compared to the isogenic mutant ΔsigB from temporal transcriptomic analysis. Table 2S. Overview of σB-dependent down regulated genes in L. monocytogenes EGD-e wild-type compared to the isogenic mutant ΔsigB from temporal transcriptomic analysis. Table 3S. Primers used in this study. Fig. 1S. Survival of L. monocytogenes EGD-e wild type as compared to isogenic deletion mutant ΔsigB during growth in BHI at pH 7.0 and at low pH of 2.5. Fig. 2S. Map of the recombinant plasmid vector pSOG30222 used for the study of promoter activities in L. monocytogenes strains. Fig. 3S. Bile tolerance assay for L. monocytogenes EGD-e and ΔsigB. Fig. 4S. Growth at 37°C in BHI of L. monocytogenes EGD-e and ΔsigB. [file 1471-2180-8-20-S1.pdf]
